# Supplementary material for: Augmenting the availability of historical GDP per capita estimates through machine learning
Source: Proc Natl Acad Sci U S A. 2024 Sep 16;121(39):e2402060121. doi: 10.1073/pnas.2402060121 (PMC11441543; doi:10.1073/pnas.2402060121)
Supplement: Supplementary file 1 — Appendix 01 (PDF) [file pnas.2402060121.sapp.pdf]

Supporting Information for:

Augmenting the availability of historical GDP per capita estimates through machine learning

Philipp Koch[1,2] , *Viktor Stojkoski*[1,3] , *César A. Hidalgo*[1,4,5]

[1] Center for Collective Learning, ANITI, IRIT, Université de Toulouse, Toulouse, France.
[2] EcoAustria – Institute for Economic Research, Vienna, Austria.
[3] Faculty of Economics, University Ss. Cyril and Methodius, Skopje, North Macedonia.
[4] Center for Collective Learning, CIAS, Corvinus University, Budapest, Hungary.
[5] Toulouse School of Economics, Université de Toulouse, Toulouse, France.

Content

1. SOURCE DATA ON GDP PER CAPITA LEVELS 2

2. GEOGRAPHY 4

2.1. REGIONAL CLASSIFICATIONS 4
2.2. SUPRANATIONAL REGIONS 5

3. DATA ON FAMOUS INDIVIDUALS 6

3.1. SUMMARY STATISTICS 6
3.2. HISTORICAL POPULARITY INDEX (HPI) 8
3.3. FAMOUS INDIVIDUALS AS PROXY FOR POPULATION LEVELS 9
3.4. MIGRATION 10

4. METHODS 11

4.1. ECONOMIC COMPLEXITY 11
4.2. SINGULAR VALUE DECOMPOSITION 15

5. RESULTS 16

5.1. EN MODEL RESULTS 16
5.2. ATLANTIC TRADE 22
5.3. GENERALIZABILITY OF THE RESULTS 23
5.4. GERMAN REGIONS AFTER THE FRENCH REVOLUTION 26
5.5. ROBUSTNESS 26
5.5.1. USING ONLY DATA PRIOR TO THE YEAR 2000 26
5.5.2. COMPARING RESULTS ACROSS LANGUAGE EDITIONS 27

5.5.3. ASSIGNMENT OF BIOGRAPHIES TO TIME PERIODS 29
5.5.4. SCALING FEATURES USING THE INVERSE HYPERBOLIC SINE FUNCTION 31
5.5.5. BACKWARD FEATURE SELECTION 32
5.5.6. USING HISTORICAL POPULARITY TO DEFINE FEATURES 33
5.5.7. REMOVING DUMMIES FOR SUPRANATIONAL REGIONS 33
5.5.8. PREDICTING GROWTH RATES 34

6. REFERENCES 35

1. Source data on GDP per capita levels

We compile several sources on GDP per capita levels:

Maddison project (1, 2) (2020 release) for country-level historical GDP per capita levels

Regional estimates of historical income levels for United Kingdom (3, 4), Sweden (5, 6), France (7, 8), Italy (9), Spain (10), Portugal (11) and Belgium (12) covering years prior to the 21st century. We match these estimates to NUTS2-regions (2021 classification).

Regional GDP per capita levels for the year 2000 from Eurostat (13), the Office for National Statistics in the UK (14), the Bureau of Economic Analysis in the United States (15), Statistics Canada (16), the State Statistics Service of Ukraine (17), Belstat in Belarus (18), and Rosstat in Russia (19).

We transform all data points to match 2011 USD PPP, matching the 2020 release of the Maddison project. In total, we obtain a dataset with 1,268 labeled observations in 50-year intervals (1300, 1350, …, 1950, 2000). All source data is reported in the published dataset.

Adaptations. We construct a dataset in 50-year intervals (1300, 1350, …, 1950, 2000). Not all observations in the historical datasets match these intervals. To increase our labeled dataset, we make slight adjustments to the source data in two forms: (1) If a source reports GDP per capita levels for e.g. 1545 and 1555, but not 1550 (Spain, for instance, in the Maddison project), we take the average GDP per capita level of 1545 and 1555 as observation in 1550. (2) If estimates in proximity (using ≤ 20 years as a rule of thumb) to a missing observation are available, we take the closest. For instance, the Maddison project provides an estimate for Belgium in 1812 but not in 1800. Then, we use the estimate for 1812 as estimate for the year 1800. Similarly, we take the regional GDP per capita estimates of 1968 provided by the Office for National Statistics (4) in the UK for 1950.

The following list describes such adaptations made to the Maddison project:

- BEL: value of 1812 used for 1800
- Value of 1820 used for 1800 for CAN, DNK, AUT, CSK, NOR, IRL
- Value of 1870 used for 1850 for BGR, HUN, IRL, ALB, CHE, ROU
- FRA: average of 1789 and 1820 used for 1800
- HRV: value of 1952 used for 1950
- IRL: value of 1913 for 1900
- SVN: value of 1952 used for 1950
- ITA: value of 1310 used for 1300
- EST: value of 1855 used for 1850

Border changes. Country borders have changed over the past centuries, which is also reflected in the Maddison project. The source materials of the Maddison project (10, 20–29) provide detailed information on which borders the respective estimates are referring to. For instance, data in the Maddison Project for Italy prior to the late 19th century refers only to Northern Italy. We take the following border changes into account when assigning biographies to geographies:

Great Britain: Data in the Maddison Project only refers to England prior to 1700. We, hence, treat England, Wales, Scotland and Northern Ireland as separate countries prior to 1700.

Netherlands: Data in the Maddison Project only refers to Holland (i.e. the NUTS-2 regions NL32 and NL33) prior to 1807.

Italy: Data in the Maddison Project only refers to Northern Italy (i.e. the NUTS regions ITC, ITH, ITI1, ITI2 and ITI3) prior to 1861.

Germany: Data in the Maddison project prior to 1850 refers to the "overlap between the Holy Roman Empire in the borders of 1792 and the territory of the nation state formed in 1871". Specifically, we take this into account by adding several regions of Poland (i.e. PL42, PL43, PL51, PL52, PL224, PL227, PL228, PL229, PL22B & PL22C) and Belgium (i.e. BE336) to Germany, while removing South Schleswig (i.e. DEF07 & DEF0C).

Poland: Data in the Maddison project prior to 1850 refers to the district of Cracow only.

Czechoslovakia: In the Maddison Project, estimates for Czechia or Slovakia do not exist prior to 1993, but just for Czechoslovakia (starting in 1820). Hence, we apply the borders of Czechoslovakia between 1820 and 1993. For earlier periods, however, we generate separate out-of-sample estimates for Czechia and Slovakia.

2. Geography

2.1. Regional classifications

We use the following geographical units for regions:

- European Union and EFTA countries: NUTS2-regions (Figure S1), 2021 edition
- Rest of Europe (BLR, UKR, RUS, BIH, MDA): oblasts or regions of similar size (Figure S1)
- United States: micropolitan and metropolitan statistical areas (Figure S2)
- Canada: Metropolitan areas (Figure S2)

The *.shp* files used in this study are provided in the replication package.

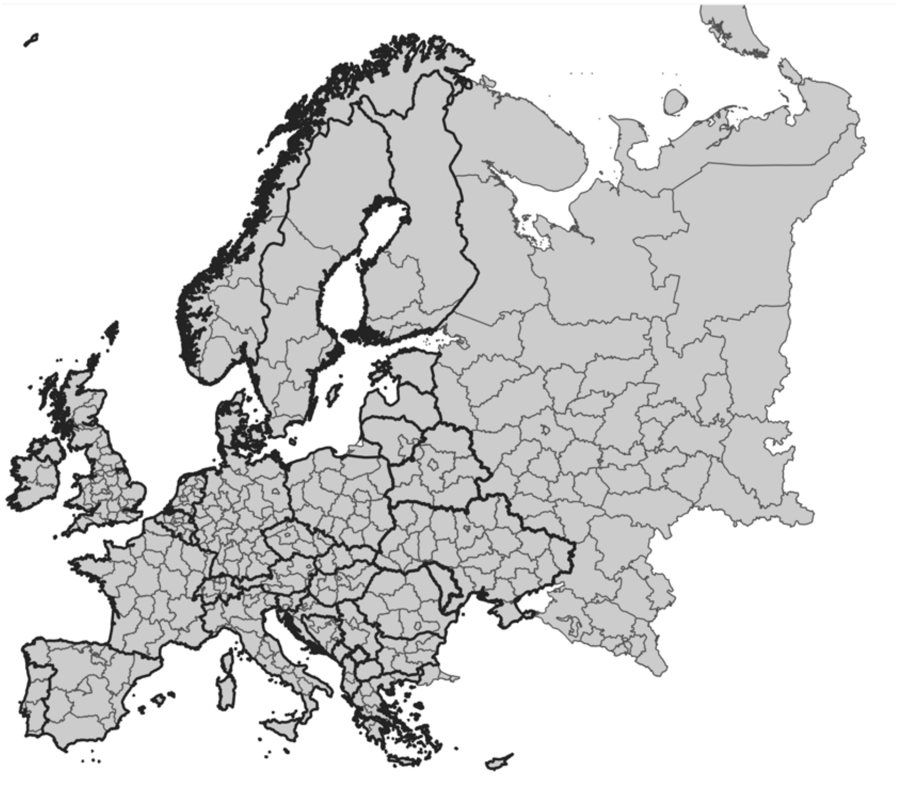

Figure S1. Administrative borders in Europe (NUTS2 regions, oblasts and similar regions)

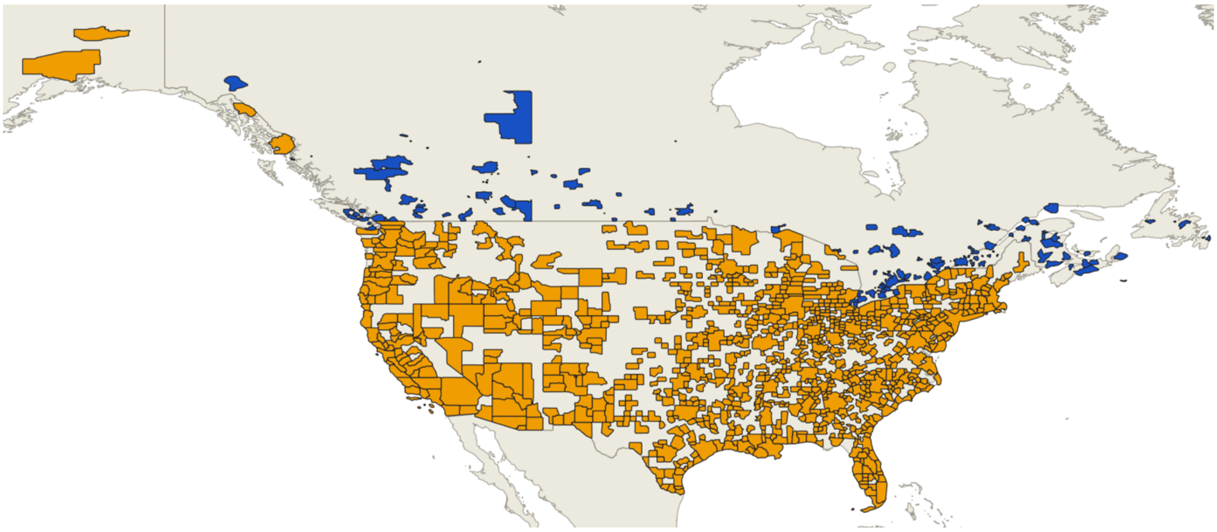

Figure S2. Administrative borders in the United States (orange; micro- and metropolitan statistical areas) and Canada (blue; metropolitan areas)

2.2. Supranational regions

We use supranational regions as fixed effects in our baseline model and potential candidates in the elastic net model. This classification mostly follows the UN geoscheme.

Specifically, we create dummy variables for the following region-time combinations. We need to aggregate early time periods due to limited observations:

- Southern Europe up to 1800
- Northern Europe up to 1800
- Western Europe before 1800
- Eastern Europe before 1800
- Northern America before 1800
- Western Europe in 1800
- Eastern Europe in 1800
- North America in 1800
- Southern Europe in 1850
- Northern Europe in 1850
- Western Europe in 1850
- Eastern Europe in 1850
- North America in 1850
- Southern Europe in 1900
- Northern Europe in 1900
- Western Europe in 1900
- Eastern Europe in 1900
- North America in 1900
- Southern Europe in 1950
- Northern Europe in 1950

- Western Europe in 1950
- Former Soviet Union in 1950
- North America in 1950
- Southern Europe in 2000
- Northern Europe in 2000
- Western Europe in 2000
- Former Soviet Union in 2000
- North America in 2000

One change we make to the UN geoscheme in assigning countries to supranational regions concern the Baltic states. We assign them to Northern Europe prior to 1750 (which they are originally in the UN geoscheme), to Eastern Europe between 1750 and 1950, and to former Soviet Union countries in 2000.

3. Data on famous individuals

We use a recently published and the most comprehensive database for notable people from Wikipedia, curated and cross-verified by Morgane Laouenan and colleagues (30). This database collects data on 2.29 million famous individuals across human history, including their places of birth and death, their occupation, and proxies of their historical importance such as Wikipedia page views or the number of language editions.

3.1. Summary statistics

We assign biographies to countries and regions and use only those biographies that satisfy the following conditions:

- Wikipedia pages in at least two language editions
- An identifiable occupation

The latter is rooted in the fact that the granular occupation classification provided by Laouenan and colleagues is imperfect. Specifically, for the 633,820 famous individuals with at least two Wikipedia editions and living in Europe or the United States between 1150 and 2000, the database shows 2,750 unique occupations, differentiating between e.g. *actor* and *actress*, *designer* and *fashion designer* or *zoologist* and *biologist*. We manually clean the occupations and derive a classification with 49 unique occupations.

Figure S3 provides a treemap of the distribution across occupations in the dataset.

Table S1 shows the unbalanced distribution of biographies across time. While the dataset includes 1.417 individuals born between 1150 and 1299, it provides information on 364.252 individuals born between 1850 and 1999.

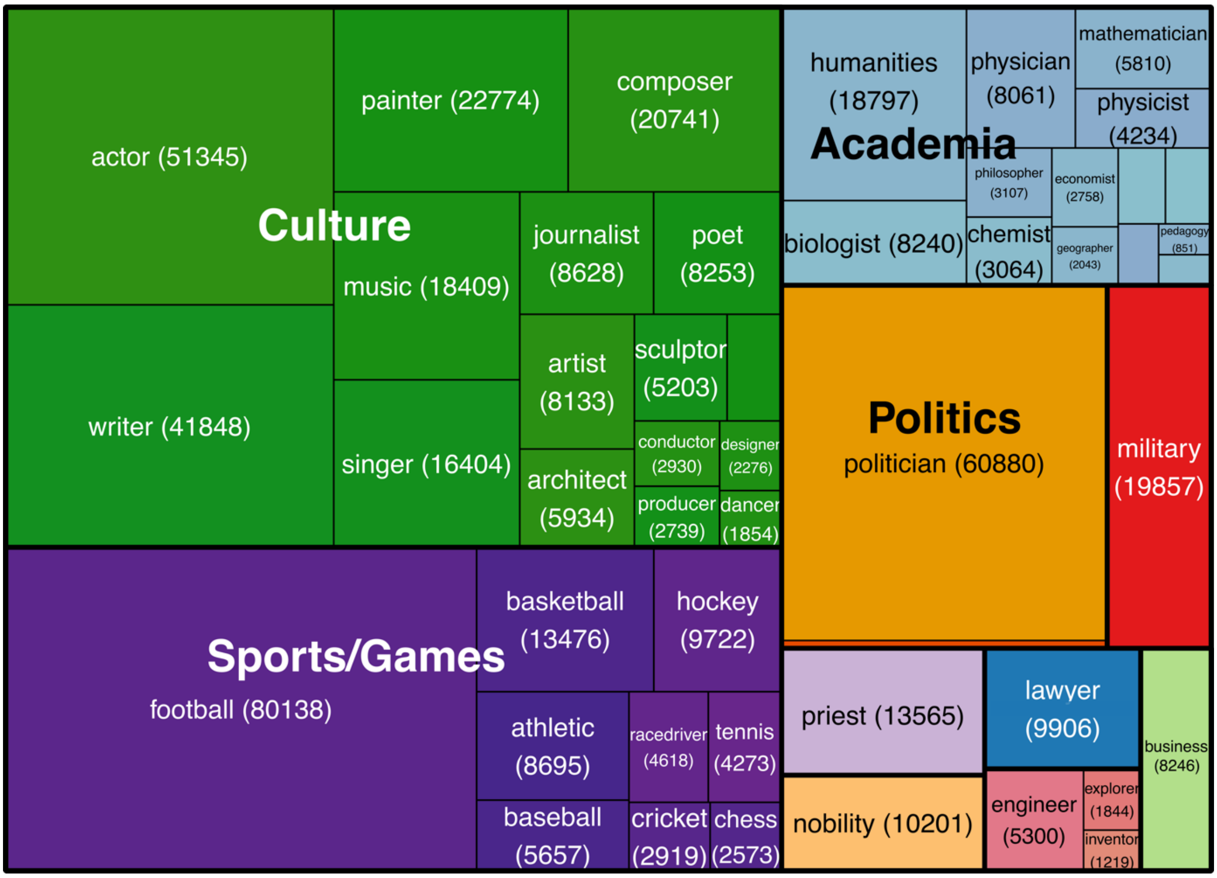

Figure S3. Treemap of occupations in the dataset on famous individuals.

Table S1. Number of famous individuals across time periods

Period	Born after	Born before	No. of observations
1	1150	1299	1.417
2	1200	1349	1.664
3	1250	1399	1.965
4	1300	1449	2.676
5	1350	1499	4.525
6	1400	1549	7.005
7	1450	1599	8.960
8	1500	1649	10.653
9	1550	1699	11.659
10	1600	1749	15.376
11	1650	1799	29.147
12	1700	1849	60.258
13	1750	1899	123.974
14	1800	1949	241.214
15	1850	1999	364.252

3.2. Historical Popularity Index (HPI)

We take the historical popularity of the individuals in our dataset into account. We follow the Historical Popularity Index (HPI) which has been introduced in the Pantheon database (31). In its original version, the HPI takes the individual's age, the number of language editions, the effective number of language editions based on the entropy in terms of page views across languages, the page views in non-English Wikipedia editions, and the variance of page views across different language editions into account.

We reconstruct the HPI with information we have available in the dataset by Laouenan and colleagues (30). Specifically, an individuals' HPI is proportional to the number of Wikipedia page views (V), the number of language editions (L) and age (A, i.e. 2023 minus year of birth):

$$HPI = \begin{cases} log_{10}(V) + ln(L) + log_4(A) & if\ A \geq 70 \\ log_{10}(V) + ln(L) + log_4(A) - \dfrac{70 - A}{7} & if\ A < 70 \end{cases}$$

To assess how similar this measure of historical importance is to the HPI in the Pantheon database, we correlate these two values for the subset of famous individuals who are present in both datasets. We find that our measure of historical importance is highly correlated with the HPI in the Pantheon dataset ($R^2 = 0.76$, see Figure S4).

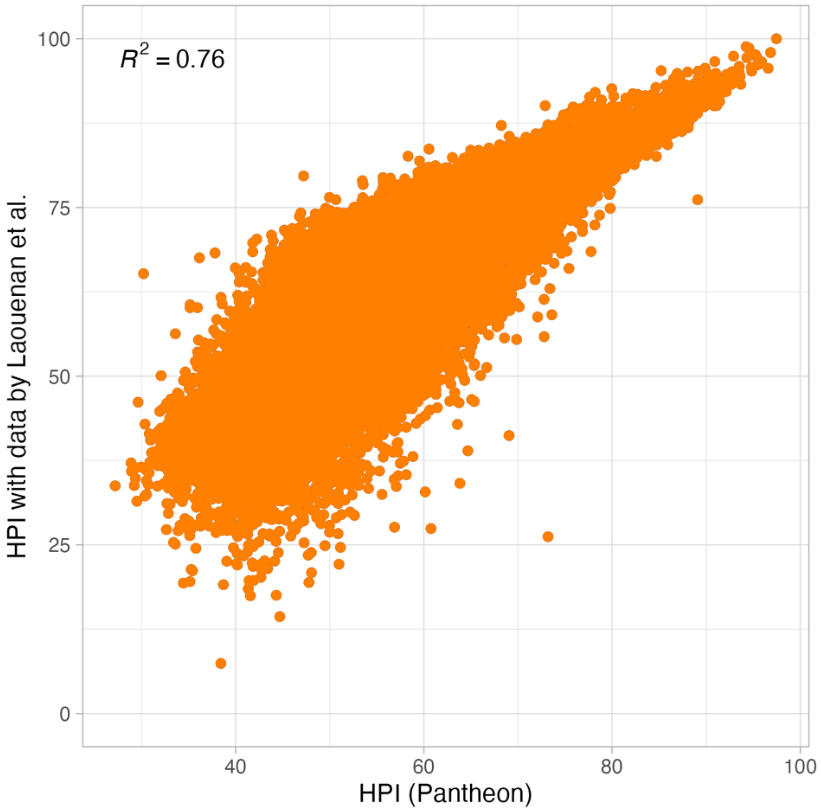

Figure S4. Correlation between the Historical Popularity Index in the Pantheon dataset and the dataset curated by Laouenan et al.

3.3. Famous individuals as proxy for population levels

Historical population data describes solely urban population (32, 33). To obtain population-weighted distributions within countries, we want to have data on population levels in rural regions as well. Hence, we use the number of famous births and deaths in a location as proxy for population. Figure S5 shows the correlation between existing data on urban population (32, 33) and the number of births and deaths in our dataset.

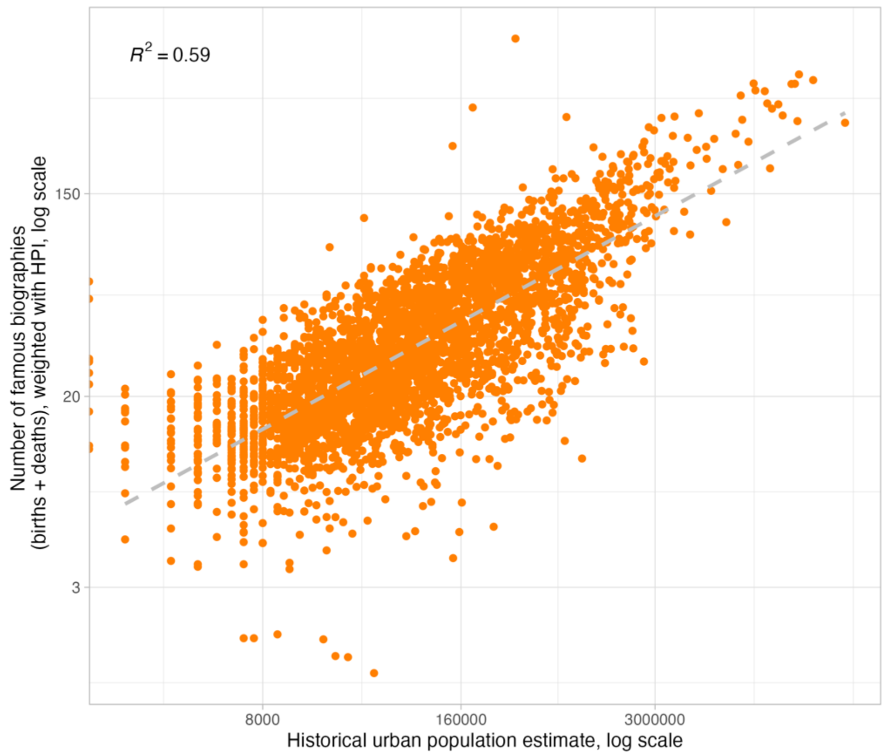

Figure S5. Correlation between historical population data and number of famous biographies in a location

3.4. Migration

We use places of birth and death as a proxy for migration, following the literature using similar data to describe migration movements (34, 35).

This evokes the question whether this proxy is valid. In a recent publication (36) we explored this question by randomly drawing ~200 individuals from the dataset, paying attention to representativeness across centuries. We read the Wikipedia article for each famous individual to determine whether a relation to the place of death exists, which would qualify as migration. We differentiated between (a) having any relation to the place of death (i.e. living there for a considerable amount of time, having noteworthy social connections with multiple visits there, or, in case of politicians and noblemen, reigning over the region) and (b) having a major relation to the place of death. The latter is the case if the place was one of the individual's main places of living, if the famous individual taught at a university there etc.

We found that in 181 out of 202 cases ($\hat{p} = 0.896$, 95% CI: [0.854, 0.938]), the famous individual had a relation to his or her place of death. Hence, only in 10% of observations the place of death is arbitrary. Also, we found that in 151 out of 202 cases ($\hat{p} = 0.748$, 95% CI: [0.688, 0.807]), the famous individual had a major relation to his or her place of death. These

results indicate that using place of birth and death as a proxy for migration is a valid approach. The sampled data is available in the GitHub repository associated with this publication (folder *misc/migration_proxy*).

4. Methods

4.1. Economic Complexity

We compute economic complexity indices for famous births, deaths, immigrants, and emigrants in a location to include them as potential features in our elastic net model. Here, we provide tables showing the 30 most complex locations in 1300 (Figure S6), 1600 (Figure S7), and 1900 (Figure S8) when considering famous births, deaths, immigrants, or emigrants.

A crucial methodological step in calculating the Economic Complexity Index is to make sure we compare locations and occupations that are not too different with respect to size. Hence, we do not compute ECI values for locations with less than eight births or deaths in a period up to 1600, with less than 20 births or deaths per period between 1650 and 1950, or with less than 50 births or deaths in 2000. For locations with fewer famous individuals, we impute the minimum ECI value.

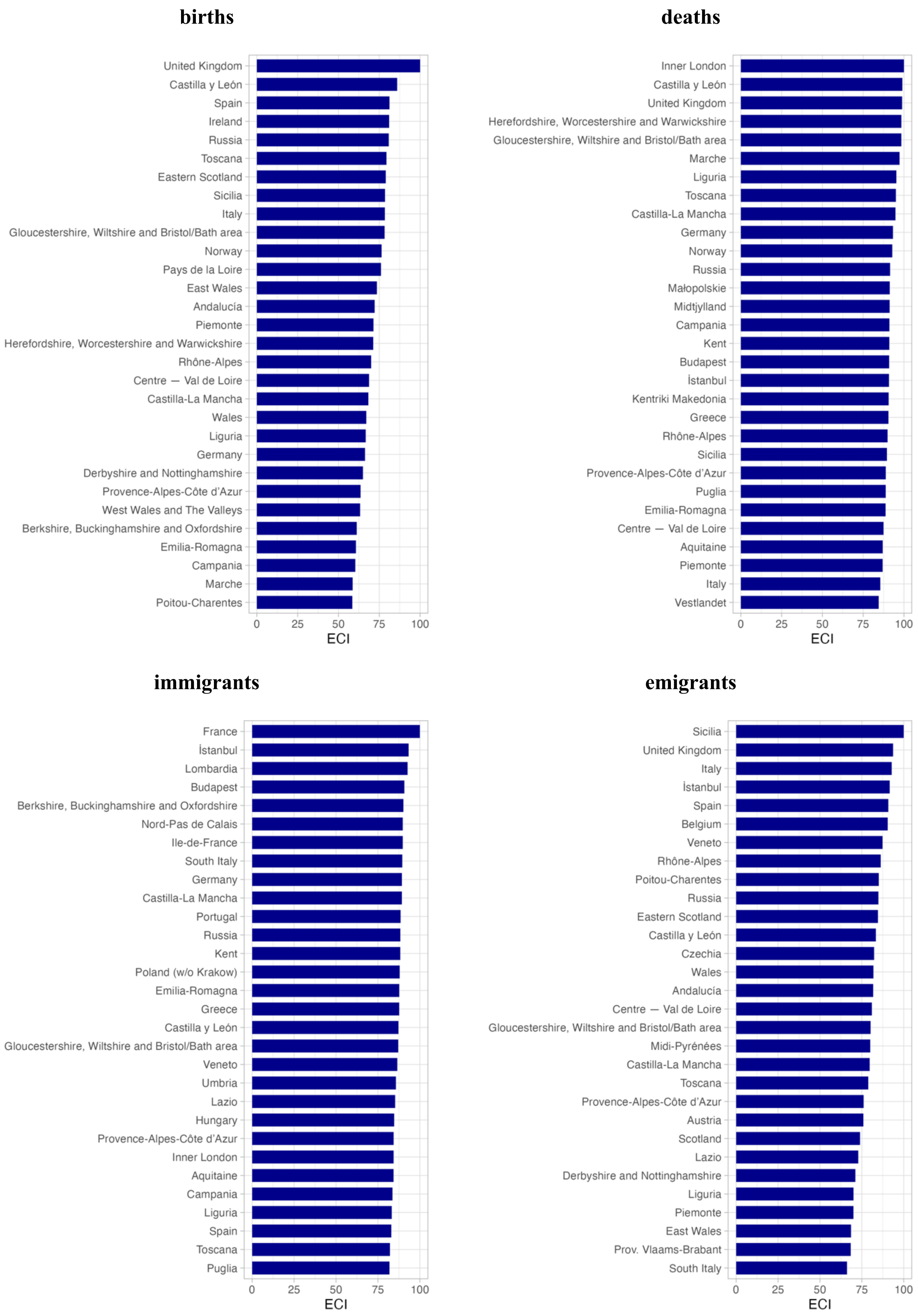

Figure S6. The 30 locations with the highest Economic Complexity Index in 1300 for births, deaths, immigrants, and emigrants.

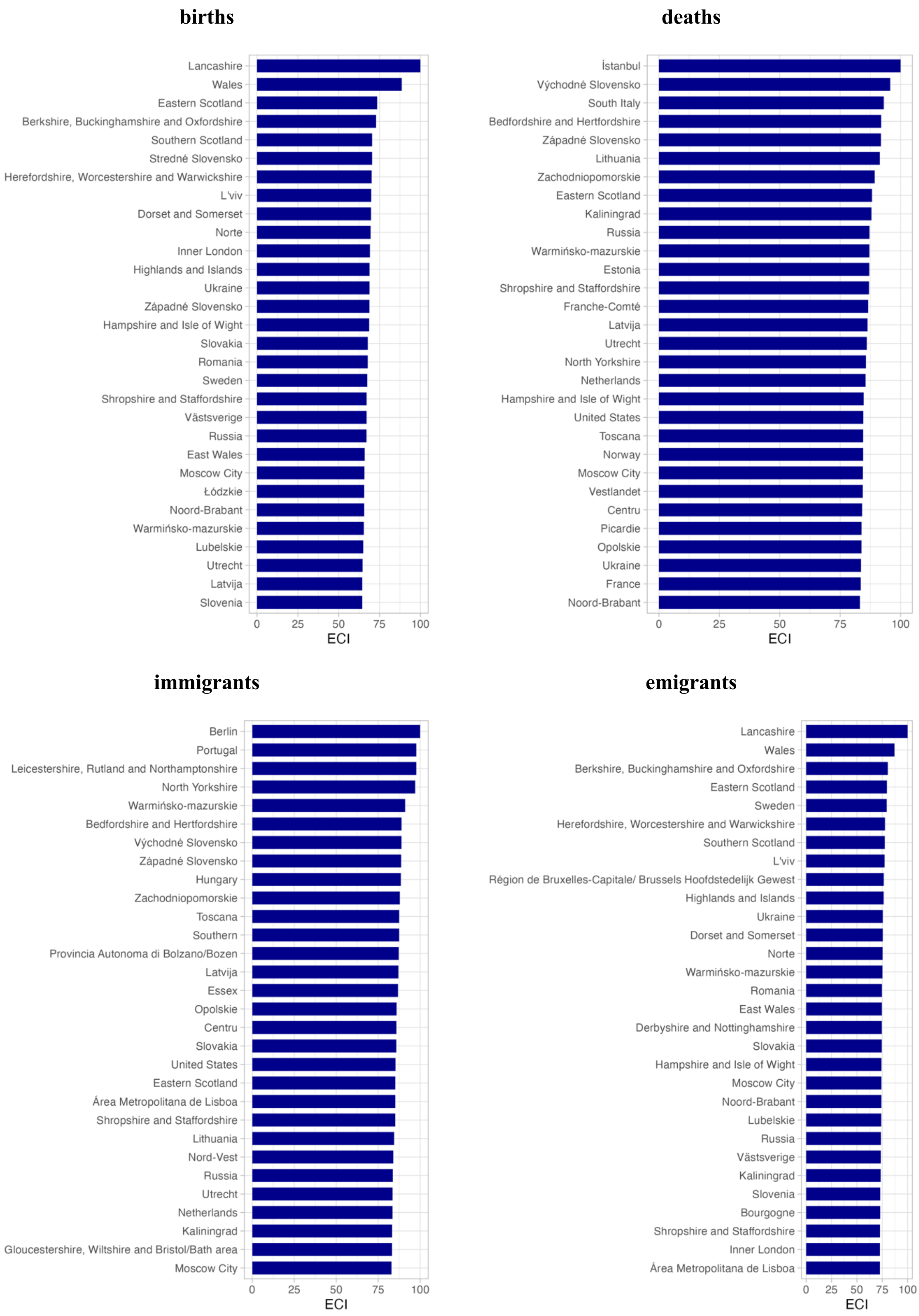

Figure S7. The 30 locations with the highest Economic Complexity Index in 1600 for births, deaths, immigrants, and emigrants.

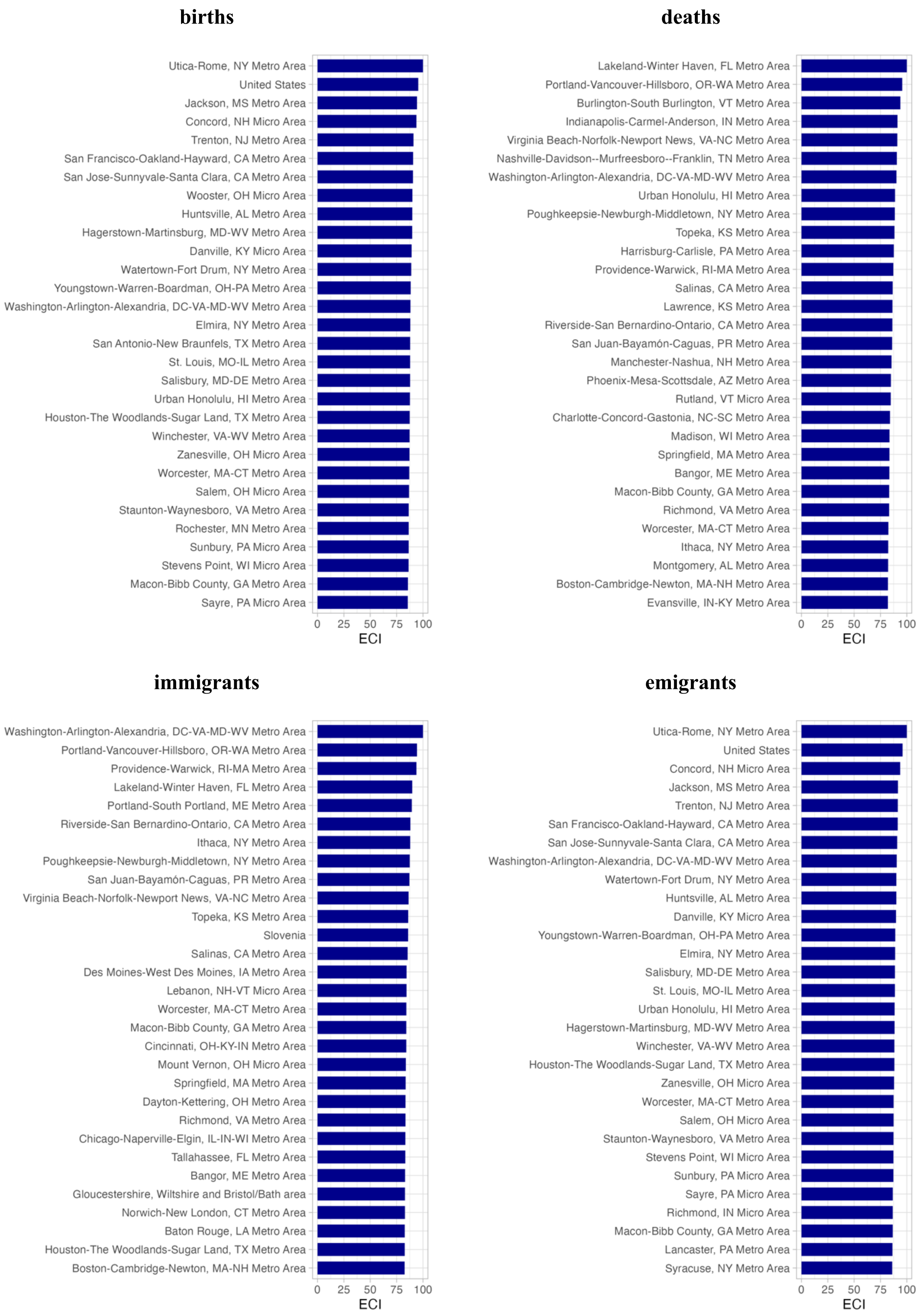

Figure S8. The 30 locations with the highest Economic Complexity Index in 1900 for births, deaths, immigrants, and emigrants.

4.2. Singular Value Decomposition

Singular Value Decomposition (SVD) is a dimensionality reduction technique which retrieves factors from rectangular matrices that best explain the structure of the underlying matrix. SVD is a generalization of the eigenvalue decomposition.

An overview of SVD and its connection to Cobb-Douglas production functions can be found in a recent review on economic complexity (37).

Two SVD factors that are selected by the elastic net model and play a significant role according to the Shapley values are the third factor of $N_{ik,1600}^{births}$ and the fourth factor of $N_{ik,1600}^{deaths}$. Figure S9 and Figure S10 plot these factors in a scatterplot with a measure of size on the vertical axis (total number of births in the location). Interpreting these factors is non-trivial. The third SVD factor of $N_{ik,1600}^{births}$ (Figure S9), for instance, seems to distinguish between some Dutch, and British regions on one side and Italian, French, and German regions on the other.

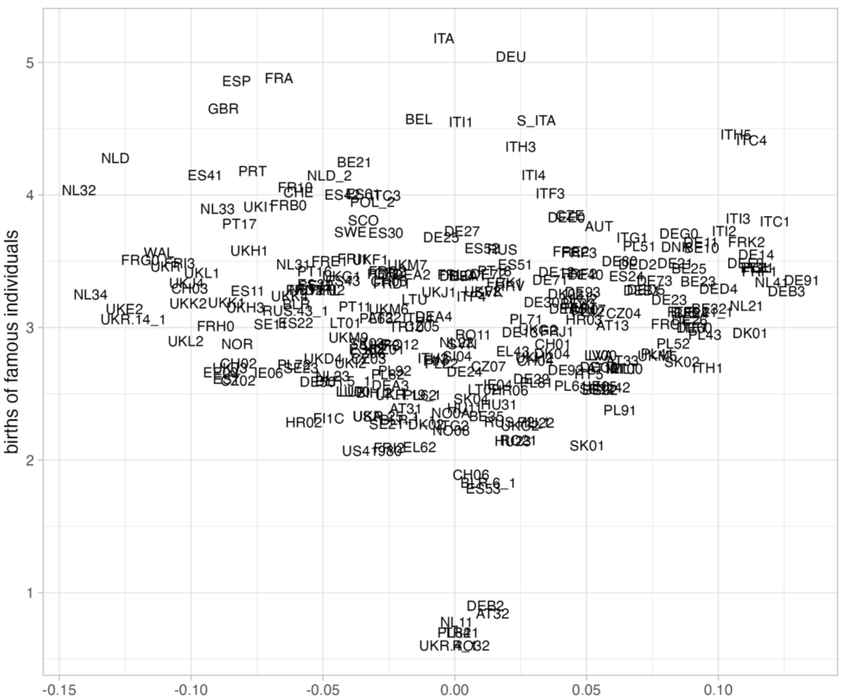

Figure S9. Third SVD factor for births, 1600

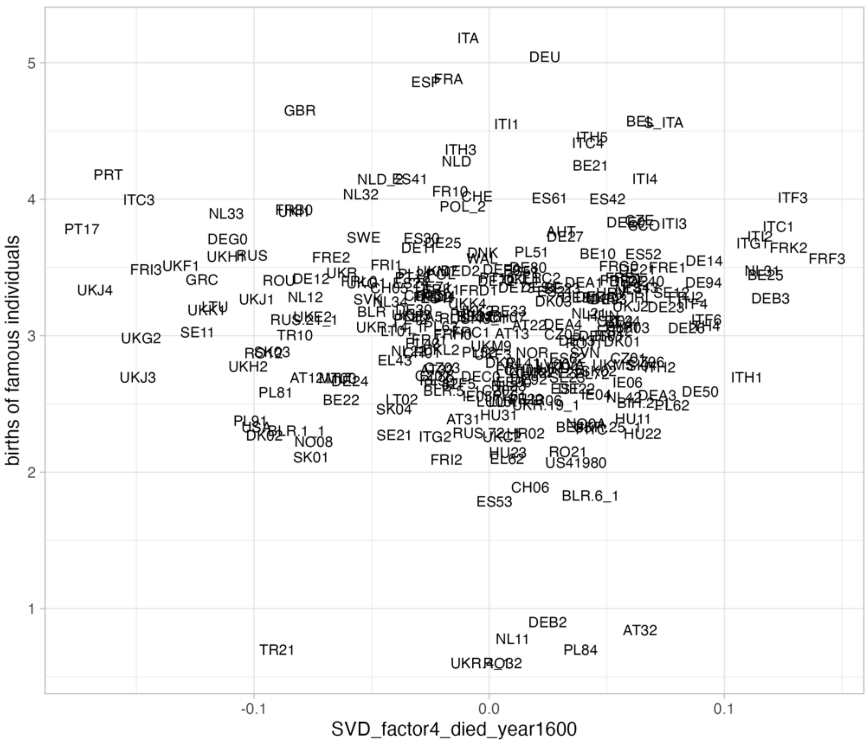

Figure S10. Fourth SVD factor for deaths, 1600

5. Results

5.1. EN model results

Here, we provide the results from the EN model for each period. This includes the plots showing the cross-validation of the model parameters as well as the selected features and their coefficients. Regularization models such as EN do not provide standard errors directly, which is why we use bootstrapping to obtain confidence intervals for our estimates. We aggregated our data to five historically informed epochs, i.e. the Late Middle Ages (1300-1500, Figure S11), the Early Modern Period (1550-1750, Figure S12), the Industrial Revolution (1800-1850, Figure S13), the Machine Age (1900-1950, Figure S14), and the Information Age (2000, Figure S15).

Also, we investigate whether the full model provides more explanatory power with respect to within-period variation, compared to the baseline model. Indeed, this seems to be the case, especially in early time periods, but the differences are relatively small (Figure S16).

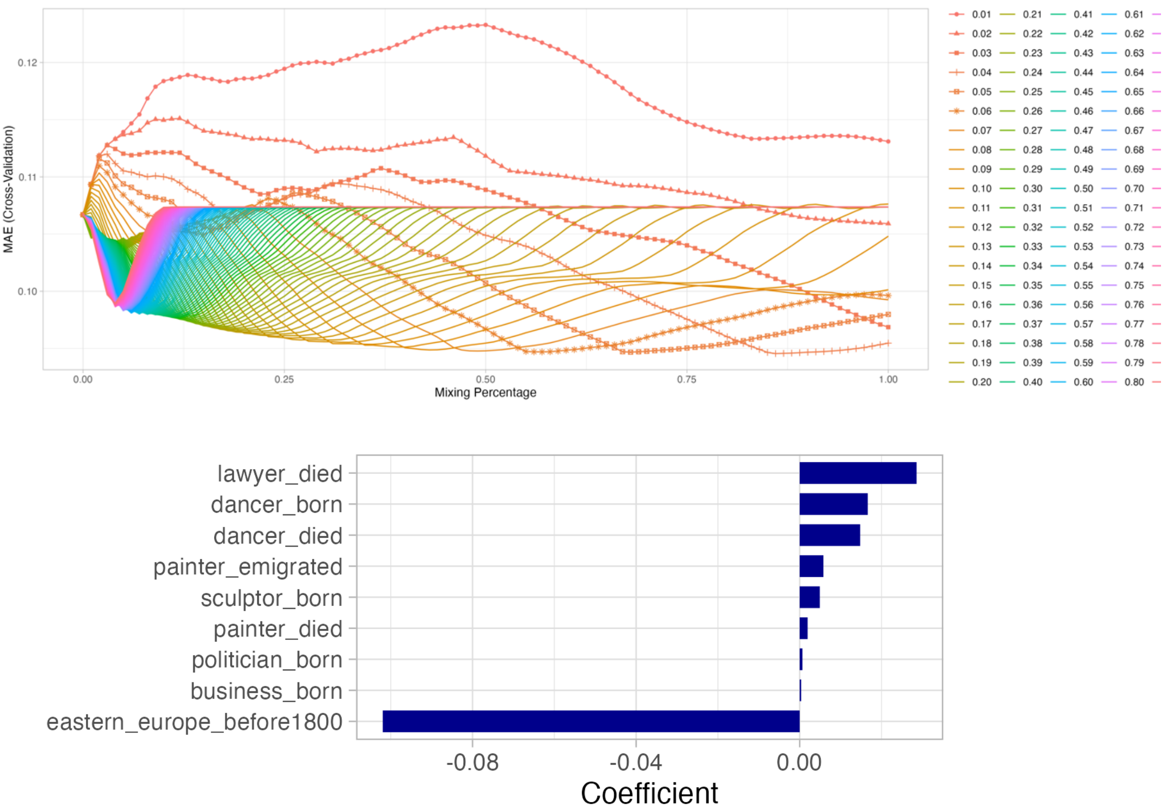

Chosen parameters:

$$\alpha = 0.86$$

$$\lambda = 0.04$$

Figure S11. Model results for Late Middle Ages (1300-1500).

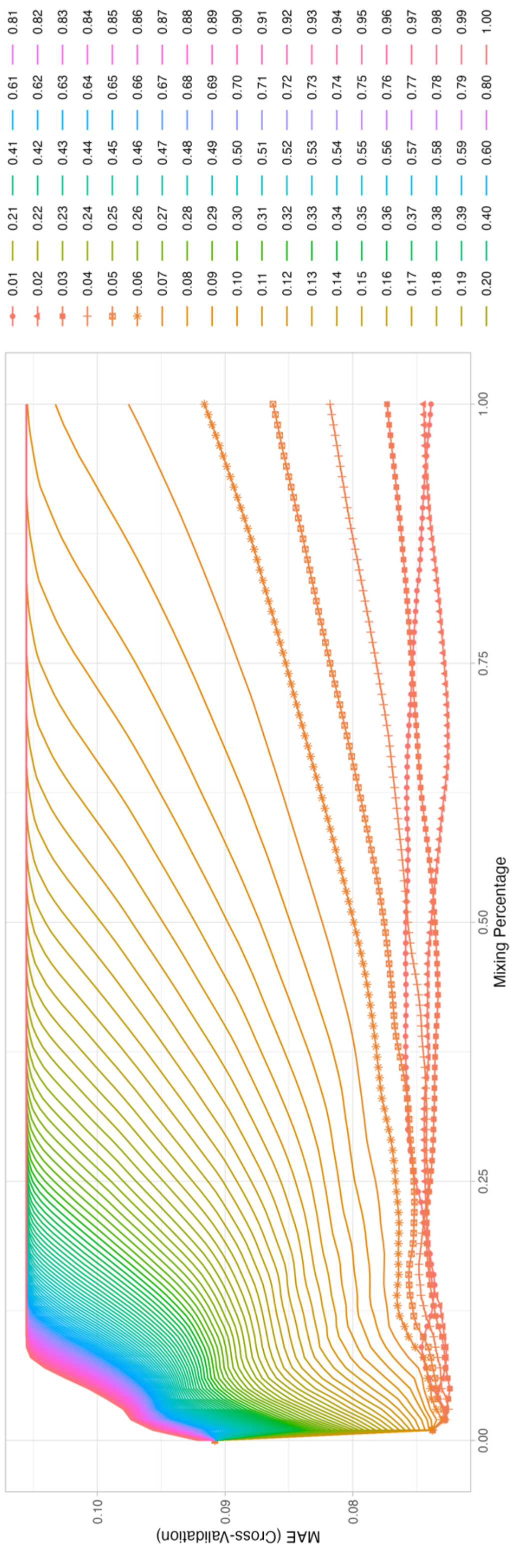

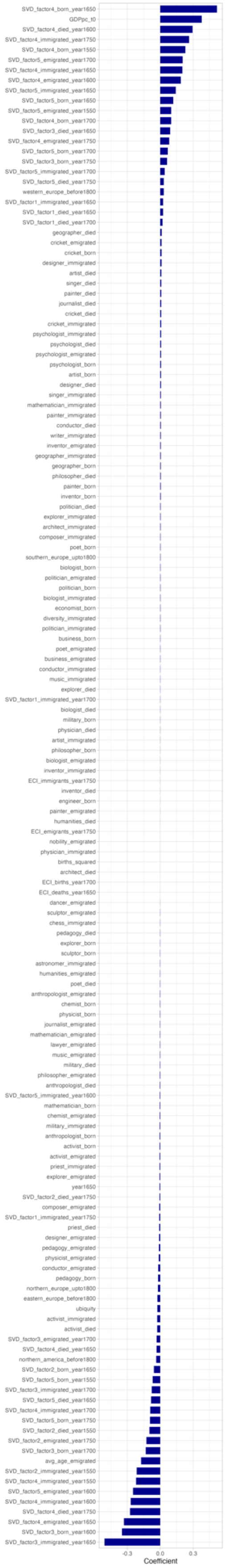

Chosen parameters:

$$\alpha = 0.05$$

$$\lambda = 0.03$$

Figure S12. Model results for Early Modern Period (1550-1750).

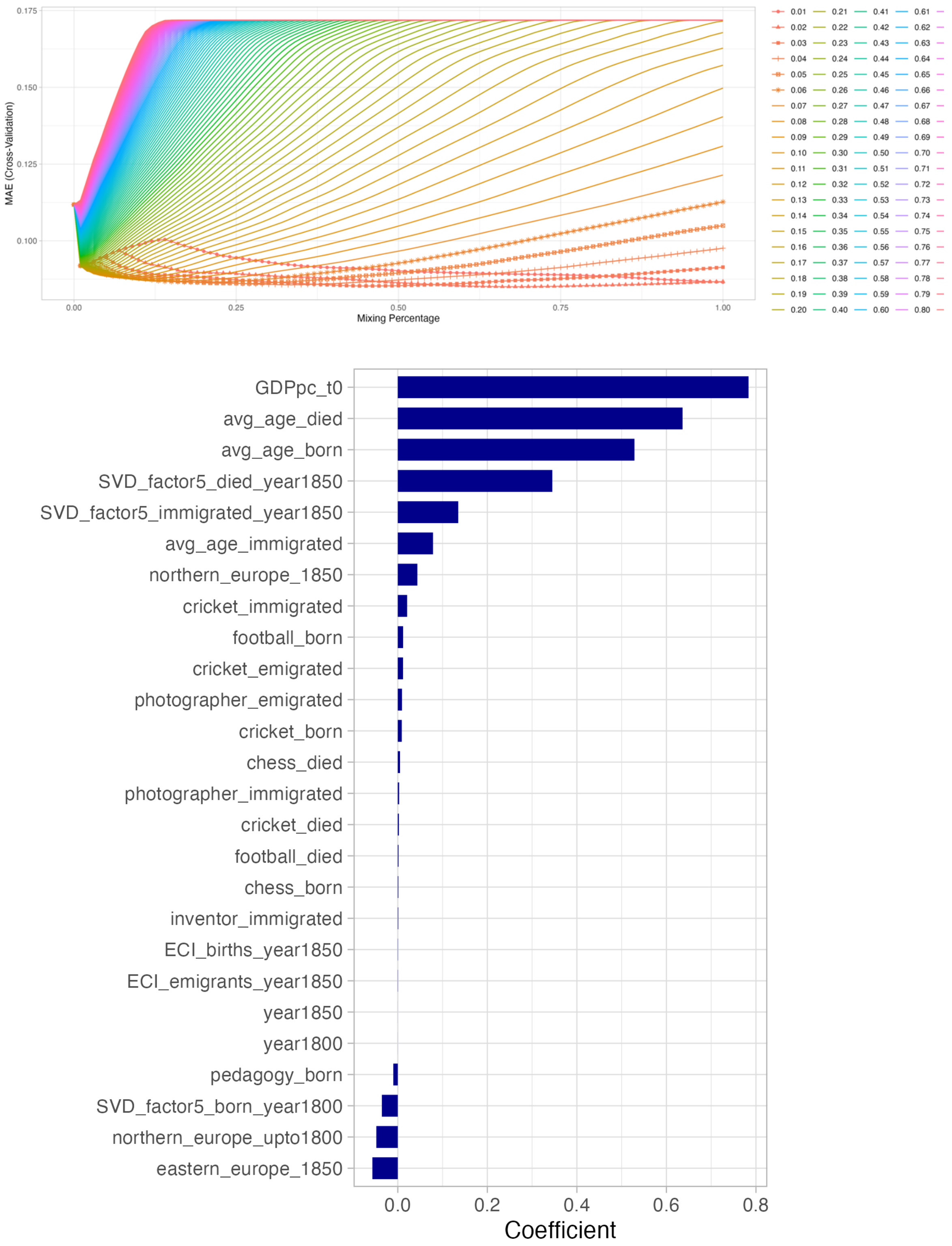

Chosen parameters:

$$\alpha = 0.68$$

$$\lambda = 0.02$$

Figure S13. Model results for Industrial Revolution (1800-1850).

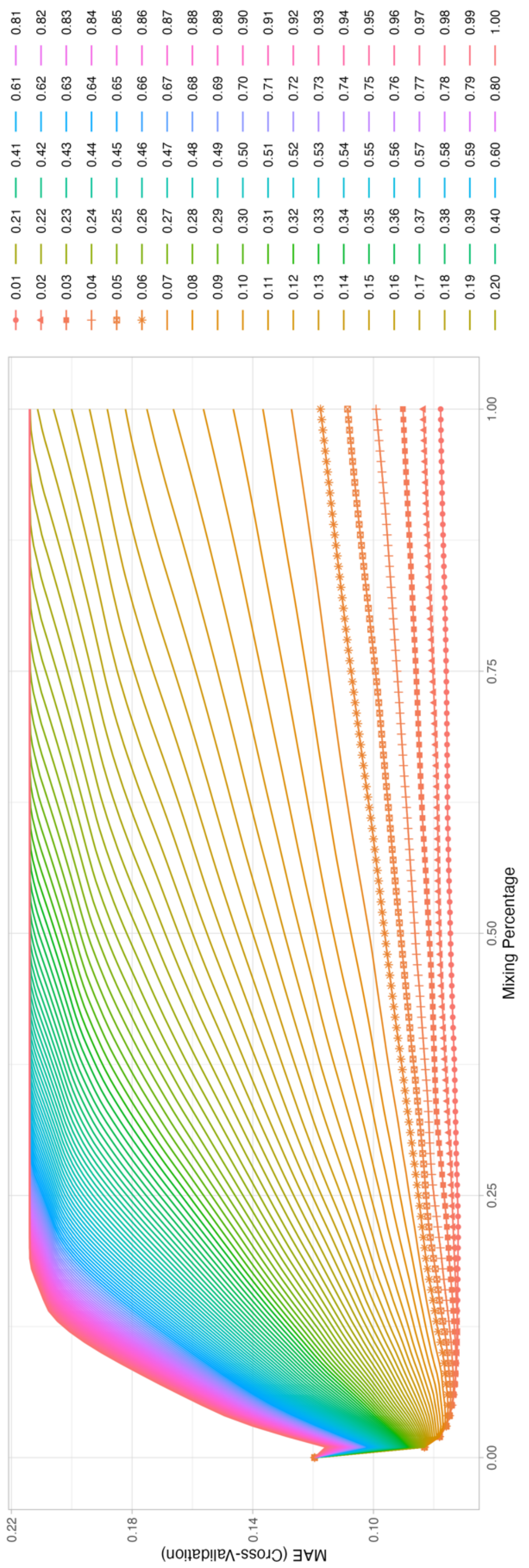

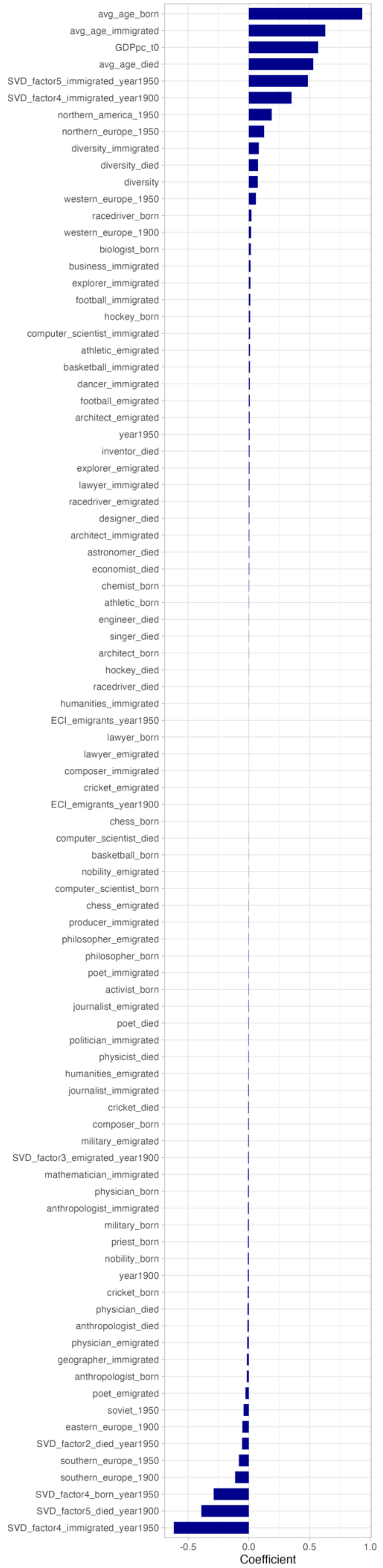

Chosen parameters:

$$\alpha = 0.22$$

$$\lambda = 0.01$$

Figure S14. Model results for Machine Age (1900-1950).

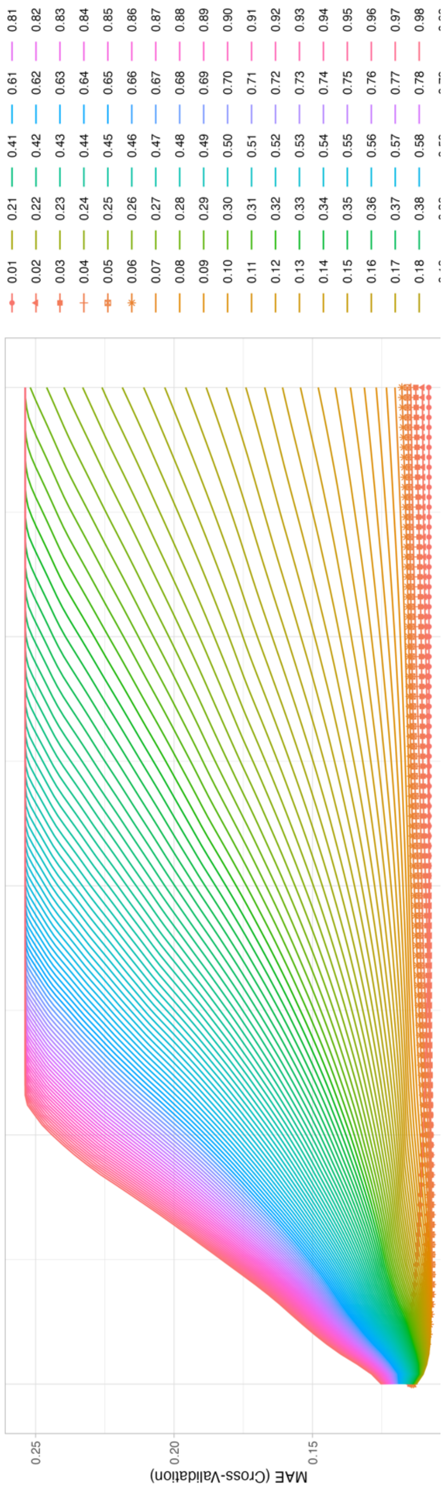

Chosen parameters:

$$\alpha = 0.1$$

$$\lambda = 0.06$$

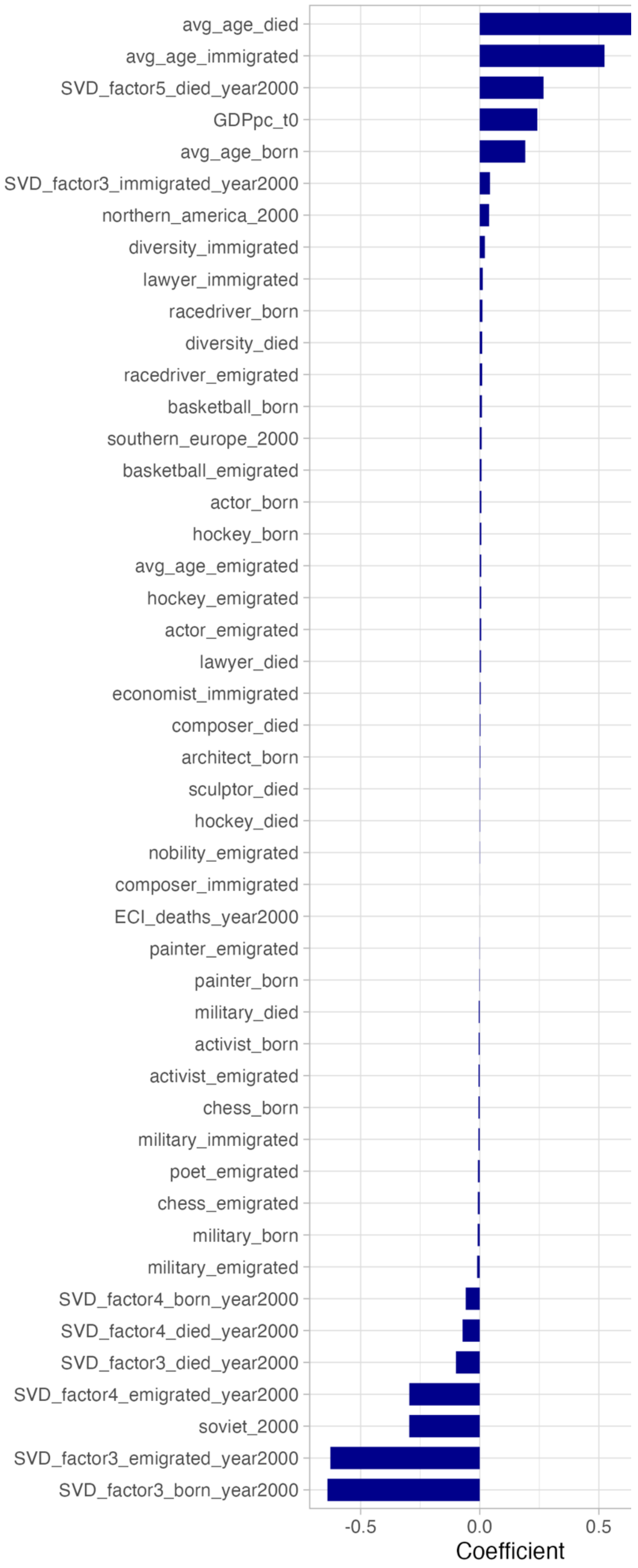

Figure S15. Model results for year 2000.

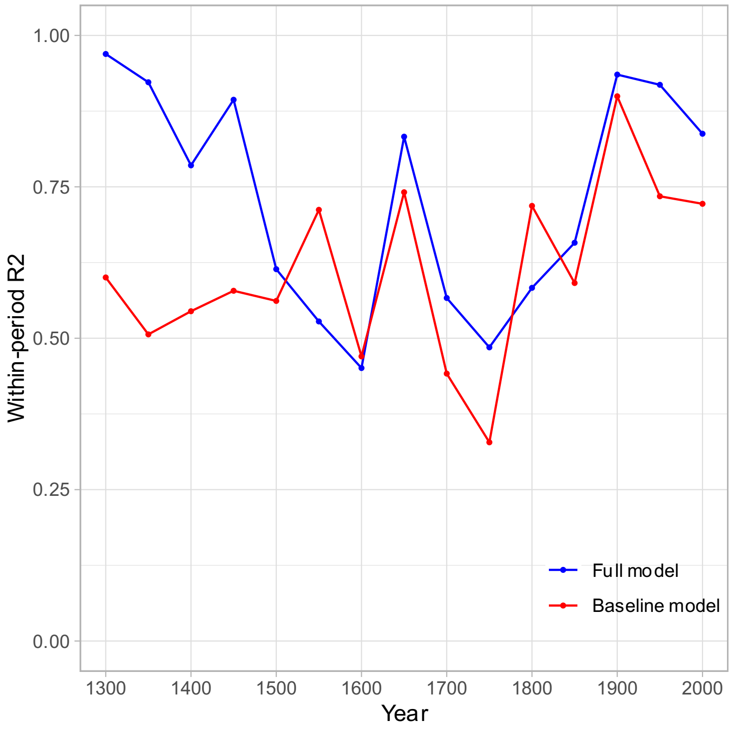

Figure S16. Explanatory power of the models within-period.

5.2. Atlantic trade

We follow the study by Acemoglu, Johnson & Robinson (38) to investigate the role of Atlantic trade in explaining the divergence between the North (UK, NLD, BEL) and the South (ITA, ESP, PRT) of Europe.

We use the information the authors provide in their paper, the appendix, and in the published data to recreate the subsets of cities and regions they identify having Atlantic and Mediterranean ports. Specifically, these are the NUTS-2 regions with Atlantic and Mediterranean ports:

- **Atlantic**: UKK4, UKI1, UKI2, UKK1, UKM8, NL32, NL33, NL34, FRD2, FRI2, ES61, ES13, PT11, PT17
- **Mediterranean**: ES62, ES52, ES51, FRJ1, FRL0, HR03, EL61, EL63, ITC2, ITC3, ITF1, ITF3, ITF4, ITG1, ITG2, ITH3, ITH4, ITH5, ITI1, ITI3, ITI4

We use the population-weighted average to aggregate these groups, using the number of births and deaths of famous individuals as a proxy for population.

Our results strongly resemble the results Acemoglu et al. present in their Fig. 2 for country-level development and Figs. 4 and 5 using city population as a proxy for regional economic development.

5.3. Generalizability of the results

We test the generalizability of our results in two ways.

First, we evaluate whether the labeled training data is different from unlabeled data with respect to some general features. Table S2 and Table S3 provide descriptive statistics of labeled and unlabeled observations for the GDP per capita levels in 2000, and the number of famous births, deaths, immigrants, and emigrants for countries and regions, respectively. Each observation in these tables refers to a country-year or region-year combination.

Second, we investigate the correlations between our estimated GDP per capita levels and proxies of economic development differentiated by labeled and unlabeled observations. Despite the differences in the descriptive statistics, we find encouraging results. That is, the correlations are highly similar for labeled and unlabeled observations (Figure S17, Figure S18, and Figure S19).

Table S2. Descriptive statistics for labeled and unlabeled country-level observations

Variable	Period	labeled observations			unlabeled observations		
		N (country-year)	*mean*	*sd*	*N (country-year)*	*mean*	*sd*
**GDPpc [2000]**	1300-1500	34	30977.6	6476.8	86	23253.6	14454.6
	1550-1750	55	30745	7312.8	106	22172.4	14346.8
	1800-1850	41	30791.5	11209.3	32	15575.6	13083
	1900-1950	52	29025.1	12996.3	25	11309	9453.8
	2000						
**births**	1300-1500	34	254.2	319.8	114	33.9	51.2
	1550-1750	55	927.9	985	129	119.8	189
	1800-1850	42	2064.4	3024	38	327.5	480.9
	1900-1950	54	7045.1	10508.6	29	1021.7	1960.1
	2000	41	10858.7	18404.5	2	321.5	340.1
**deaths**	1300-1500	34	187.9	235.4	114	32.5	60.6
	1550-1750	55	722.9	793.6	129	126	247.8
	1800-1850	42	1790.1	2790.1	38	311.9	536
	1900-1950	54	5373.1	8789.4	29	565.4	1568.7
	2000	41	3885.5	7855.5	2	34.5	17.7
**immigrants**	1300-1500	34	100.7	101.1	114	22.5	43.1
	1550-1750	55	412.3	478	129	88.2	166.8
	1800-1850	42	1190.5	1968.1	38	208.5	380.2
	1900-1950	54	3886.9	7150.8	29	402.5	1253.4
	2000	41	2845.7	6496.7	2	13.5	0.7
**emigrants**	1300-1500	34	167.2	177.2	114	23.9	33
	1550-1750	55	617.5	648	129	82	111.5
	1800-1850	42	1465	2195.7	38	223.9	330.3
	1900-1950	54	5558.8	8888.4	29	859	1663.1
	2000	41	9818.9	17046.3	2	300.5	321.7

Table S3. Descriptive statistics for labeled and unlabeled regional observations

Variable	Period	labeled observations			unlabeled observations		
		N (region-year)	*mean*	*sd*	*N (region-year)*	*mean*	*sd*
**GDPpc [2000]**	1300-1500	0	NaN		656	28200.5	10903.3
	1550-1750	43	25890.9	6727.9	1083	27094.9	12788.3
	1800-1850	111	29028.3	8602.1	687	28566.1	15970.2
	1900-1950	166	30714.7	9985.2	1179	30762.8	16196.8
	2000						
**births**	1300-1500	0	NaN		656	17.7	32.5
	1550-1750	43	78.8	69.6	1093	56	91
	1800-1850	111	249.1	376.5	767	89	140
	1900-1950	166	529.2	657.9	1488	207.3	501.8
	2000	790	537.6	1050.9	48	169.7	281.9
**deaths**	1300-1500	0	NaN		656	18.1	29.6
	1550-1750	43	65.2	78.8	1093	52.2	126.6
	1800-1850	111	228.4	717.6	767	79.8	217
	1900-1950	166	374.9	701.1	1488	166	631.1
	2000	790	201.2	647.8	48	40.9	51.2
**immigrants**	1300-1500	0	NaN		656	12	17.6
	1550-1750	43	44.1	61.1	1093	33	82.6
	1800-1850	111	147.4	494.9	767	53.6	152.9
	1900-1950	166	251	522.5	1488	122.5	478.5
	2000	790	147.7	505.3	48	33.6	42.5
**emigrants**	1300-1500	0	NaN		656	11.6	16
	1550-1750	43	57.7	51.6	1093	36.8	43.9
	1800-1850	111	168.1	173.9	767	62.9	81.2
	1900-1950	166	405.4	487.1	1488	163.9	356.6
	2000	790	484.1	902.3	48	162.4	270.1

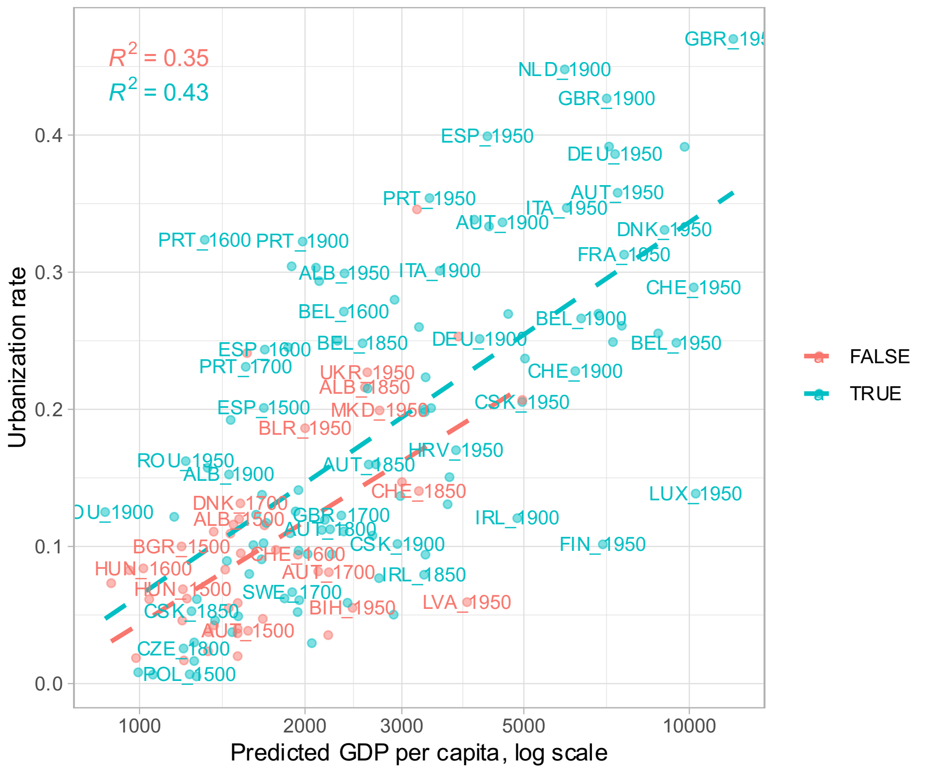

Figure S17. Correlation between estimated GDP per capita and urbanization, for labeled (TRUE) and unlabeled (FALSE) observations

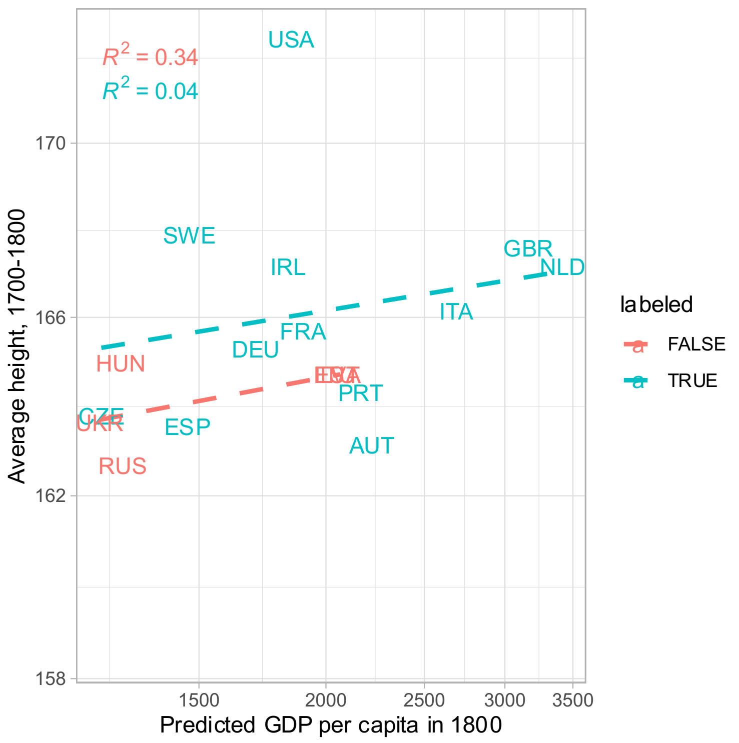

Figure S18. Correlation between estimated GDP per capita and average body height, for labeled (TRUE) and unlabeled (FALSE) observations

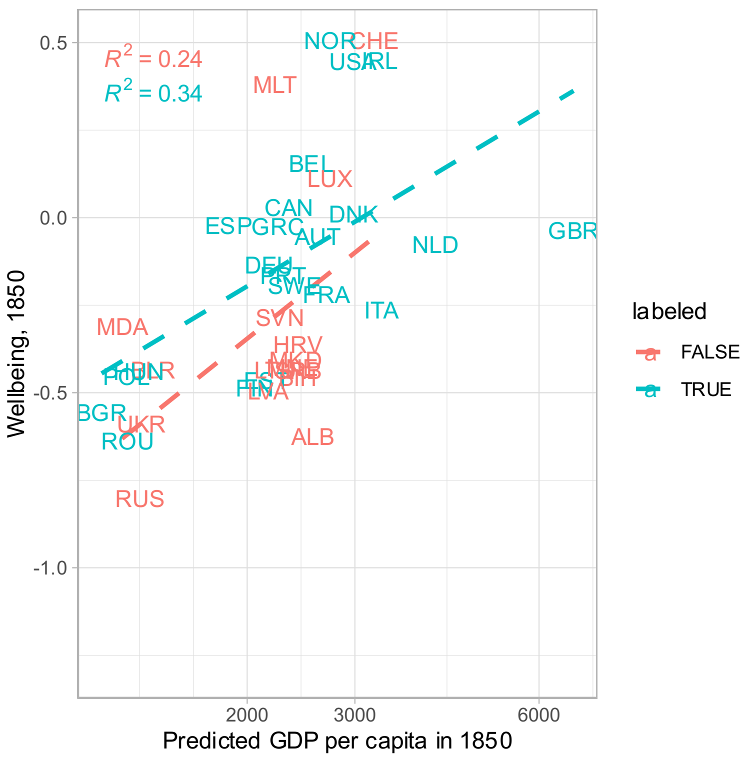

Figure S19. Correlation between estimated GDP per capita and the OECD wellbeing indicator, for labeled (TRUE) and unlabeled (FALSE) observations

5.4. German regions after the French Revolution

We explore whether our estimates replicate results by Acemoglu and coauthors regarding the economic development of German regions after the French Revolution (39).

Following the replication package the authors provide, we identify the NUTS-2 regions the 19 cities they are describing are in. Then, we compare the development of the treated group and control group the authors define in Table 1.

- Treated group: "DEB1", "DEB3", "DEA5", "DEA3", "DE91", "DEE0", "DE73", "DE92"
- Control group: "DE12", "DE21", "DE71", "DED2", "DE11", "DE40", "RUS.21_1", "PL42", "PL51", "DE80", "DEF0"

We use the population-weighted average to aggregate these groups, using the number of births and deaths of famous individuals as a proxy for population.

Our results (Figure S20) strongly resemble theirs using urbanization as a proxy for economic development (see their Fig. 2B for comparison).

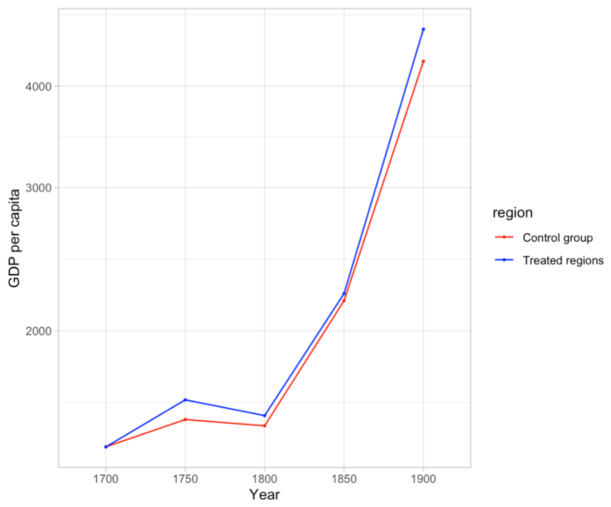

Figure S20. Economic development in German regions occupied by the French army (treated) and other German regions (control)

5.5. Robustness

5.5.1. Using only data prior to the year 2000

Our model performance results might be driven by the fact that our model is significantly better at predicting GDP per capita levels in the year 2000 than for other periods. Here, we exclude all observations of the year 2000 and rerun our model.

Indeed, model performance in terms of R-squared goes down. But the model performance in terms of the mean absolute error does not decrease.

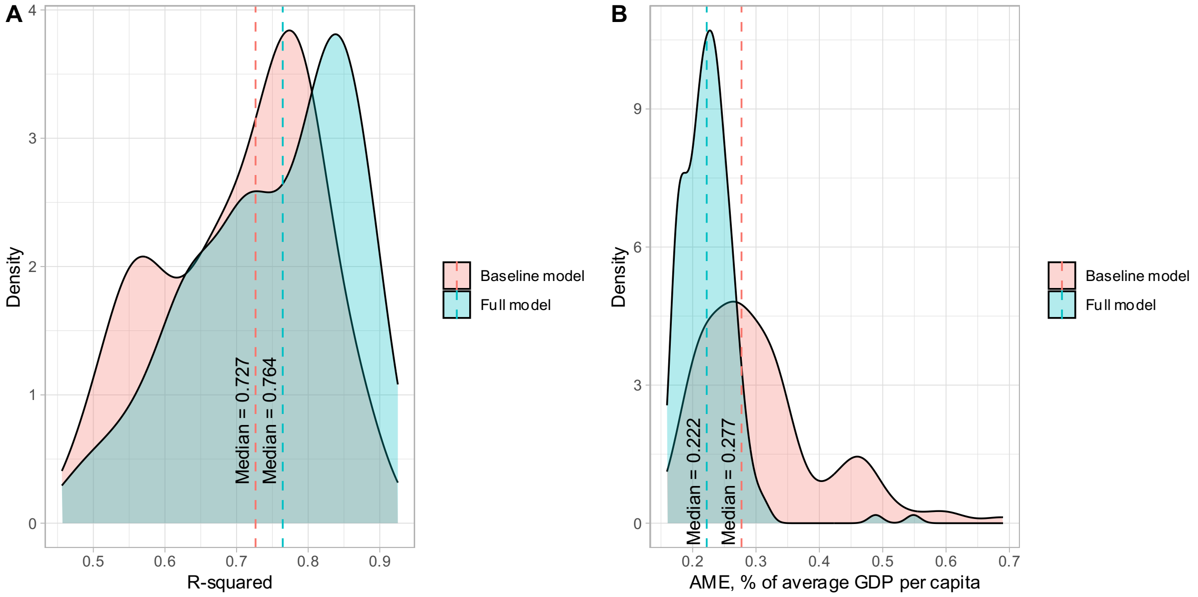

Figure S21. Model performance observations prior to the year 2000.

5.5.2. Comparing results across language editions

Wikipedia is known to have several biases, as discussed in the main manuscript. This includes an English bias. Since English Wikipedia is more comprehensive, individuals living in English speaking countries might be overrepresented. We address this issue by reducing our sample to biographies with Wikipedia pages in at least two language editions. This should reduce noise and limit the overrepresentation of biographies in English-speaking countries.

Also, we check whether our estimates are subject to an English bias by comparing our results to two other approaches of defining the sample: (1) using only pages that exist in English, and (2) using only non-English pages. If our estimates are prone to an English bias, we should observe substantial differences between the three estimates.

This, however, is not the case. Figure S22 shows the correlation of estimates obtained with these three samples of individuals. They are highly similar with a correlation coefficient of at least 0.978. We would expect the largest differences comparing estimates for English-speaking countries. Figure S23 compares estimates for US regions based on the three samples and shows that they are highly similar as well, with a correlation coefficient of at least 0.951.

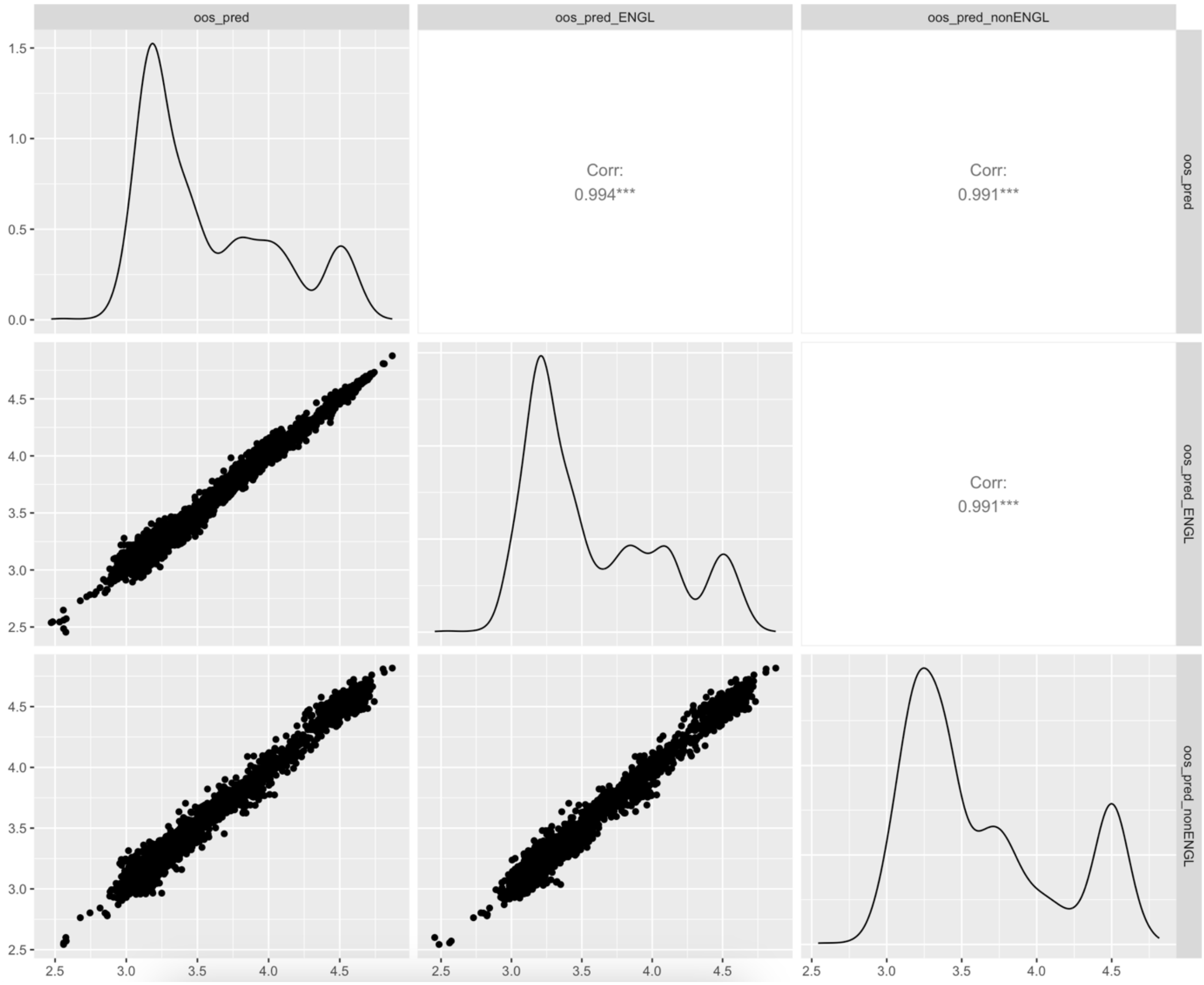

Figure S22. Comparison between estimates using biographies with at least two language editions, only English pages, and only non-English pages.

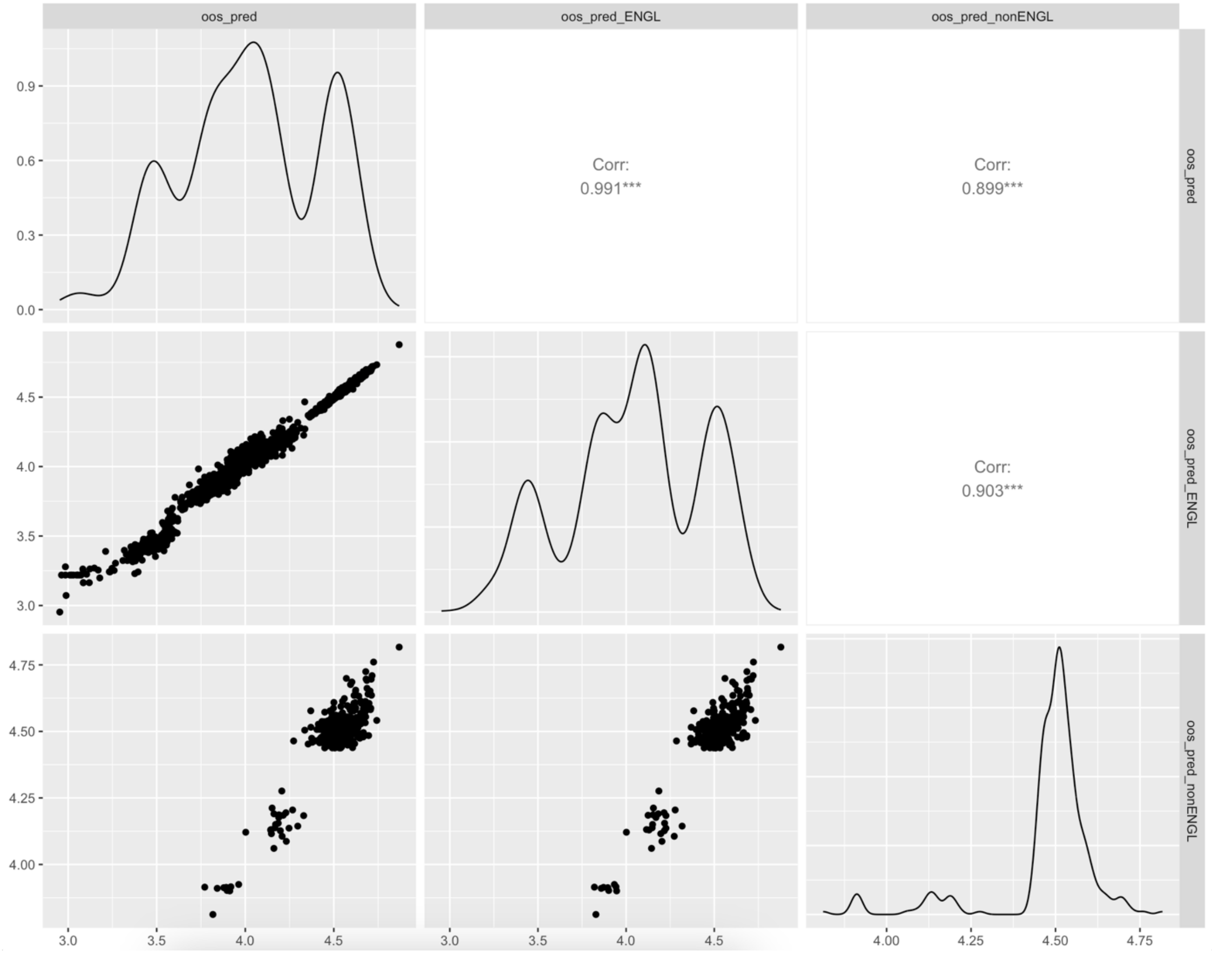

Figure S23. Comparison of estimates for US metro- and micropolitan areas using biographies with at least two language editions, only English pages, and only non-English pages.

5.5.3. Assignment of biographies to time periods

We assign individuals to time periods when they are born 150 prior to a certain year. Here, we test whether other threshold values yield different results. Specifically, we are investigating the thresholds 75 years (Figure S24), 100 years (Figure S25), and 175 years (Figure S26).

While all thresholds lead to an improvement in the out-of-sample estimates, none yields better results in terms of R^2 and absolute mean error than the model using 150 years as threshold (Fig. 2C-D in the main manuscript).

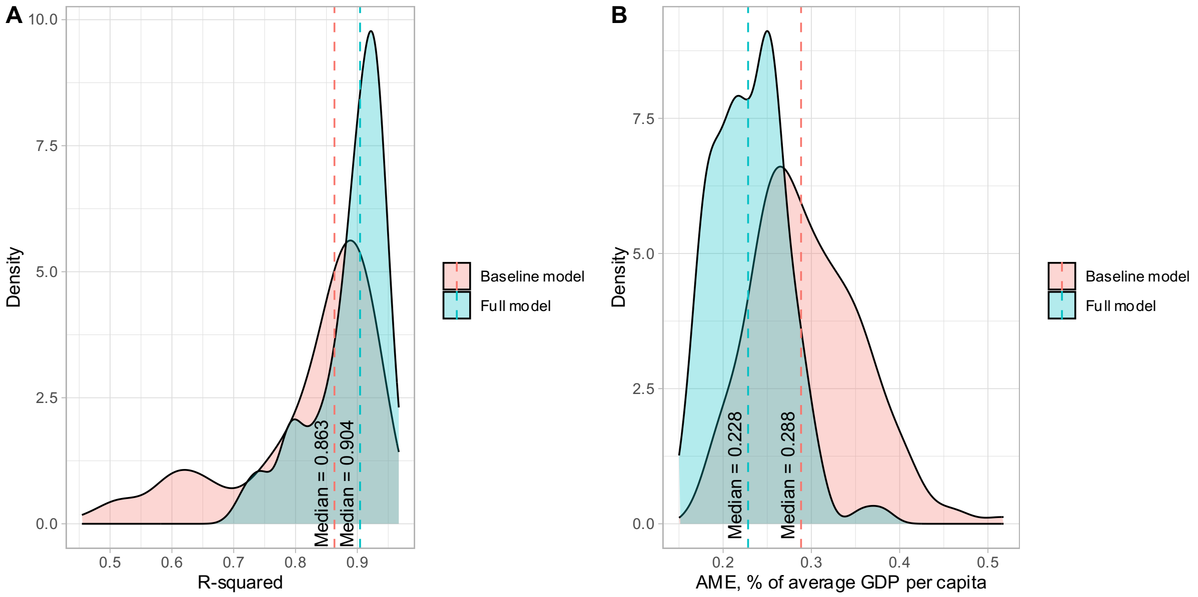

Figure S24. Model performance using individuals born 75 years prior to a certain date for extracting features.

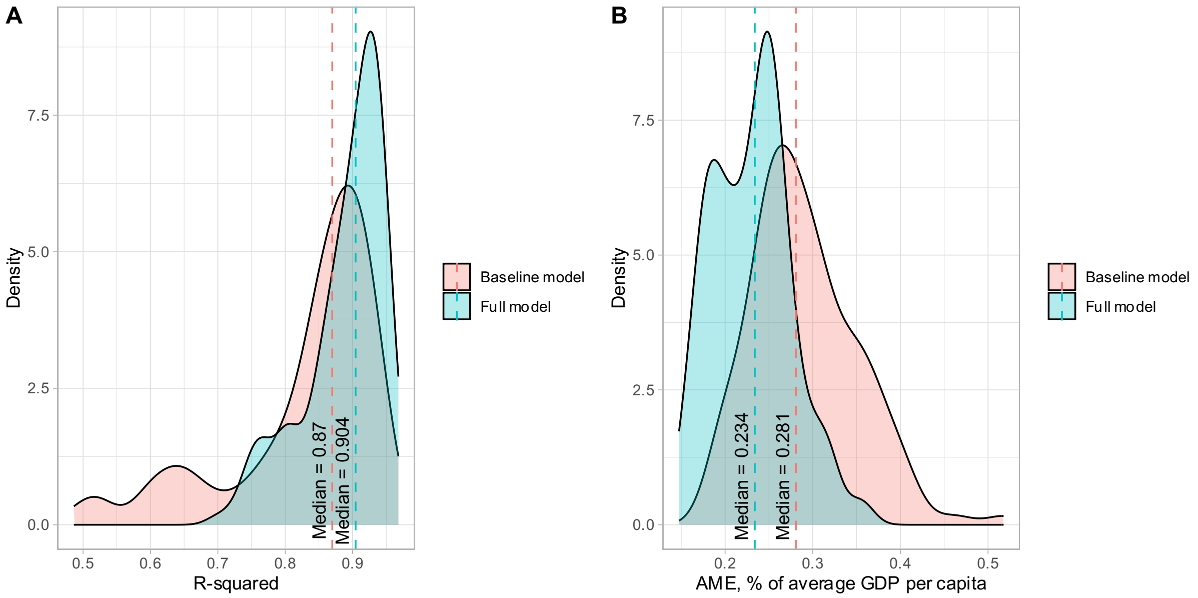

Figure S25. Model performance using individuals born 100 years prior to a certain date for extracting features.

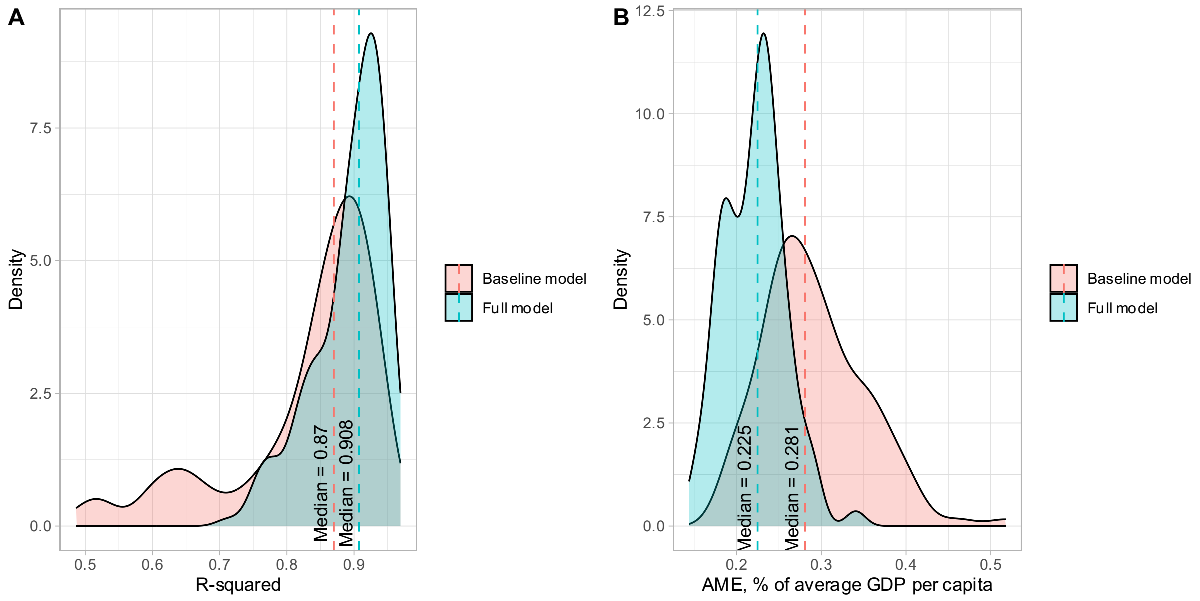

Figure S26. Model performance using individuals born 175 years prior to a certain date for extracting features.

5.5.4. Scaling features using the inverse hyperbolic sine function

We are scaling our features using logarithms. Specifically, we are using the function $\log(1 + x)$ to incorporate zeros. The inverse hyperbolic sine function is another approach that serves the same purpose. To show that our results are independent from our choice of the scaling function, we run our model using the inverse hyperbolic sine function. The model performance is very similar to using logarithms. While the R-squared is slightly better, the mean absolute error of the predictions is slightly worse (Figure S27).

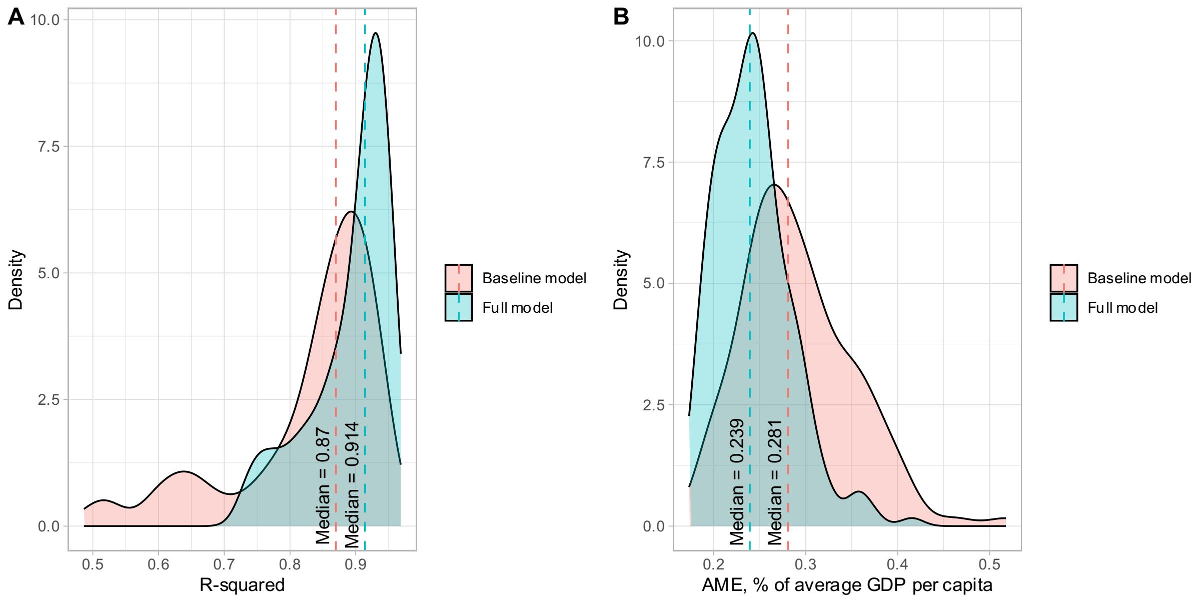

Figure S27. Model performance using inverse hyperbolic sine function to scale features.

5.5.5. Backward feature selection

We use a regularization technique, i.e. elastic net models, to select relevant features. Wrapper methods, such as backward feature selection, are also frequently used for this purpose. Here, we assess the model performance if using backward feature selection.

Backward feature selection works by recursively training the model with different subsets of features. Initially, all features are considered, and the model's performance is evaluated using k-fold cross validation. The least important feature (with respect to a feature's predictive power) is then eliminated, and the model is trained again with the reduced feature set. This process is repeated iteratively.

While we do not (need to) assume any fixed variables in the elastic net model, backward feature selection provided highly inaccurate results if no fixed variables were provided. Hence, we use the backward feature selection on top of our naïve baseline model. That is, we do not train the model with GDP per capita estimates, but with the residuals of regressing GDP per capita estimates on our baseline model. Even with this assumption, backward feature selection does not beat the baseline model in predicting the outcomes of independent test data sets with respect to the R-squared. Also, the model performance regarding the mean absolute error is lower (Figure S28).

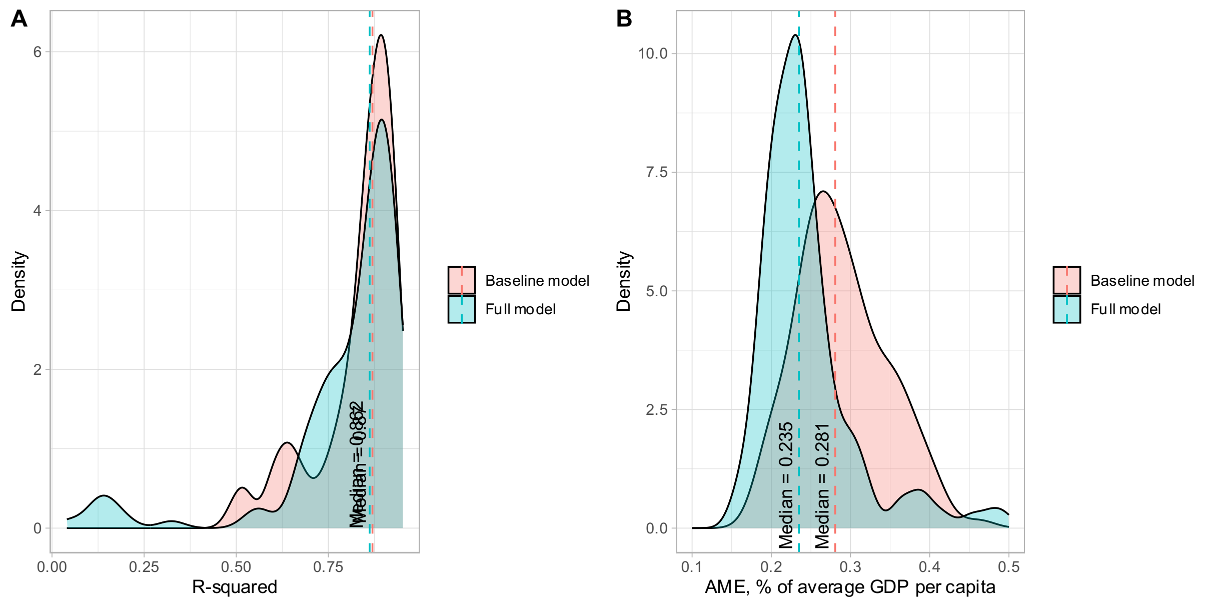

Figure S28. Model performance using backward feature selection

5.5.6. Using historical popularity to define features

In our main results, we use the Historical Popularity Index (HPI) as weights when defining features. Not using the HPI yields very similar model performance metrics (Figure S29).

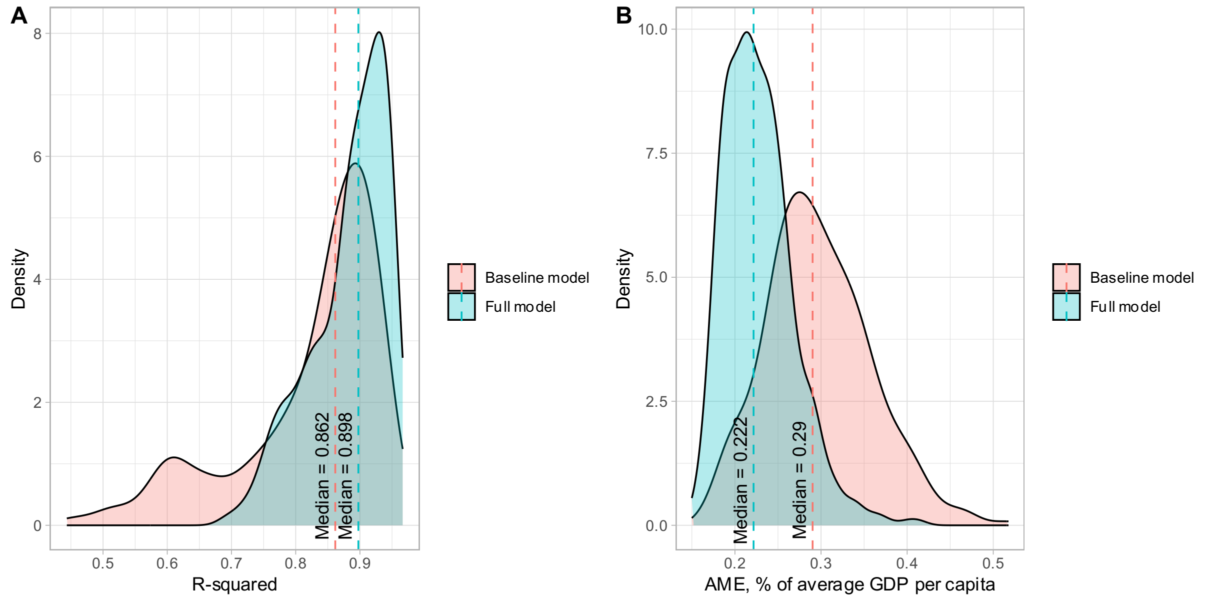

Figure S29. Model performance when not using the HPI to weigh features.

5.5.7. Removing dummies for supranational regions

We test whether the dummies for supranational regions are to a large extent driving our model performance results by removing them from the features the model can select. We find it provides highly similar results (Figure S30). These models still include the GDP per capita at the end of the previous period.

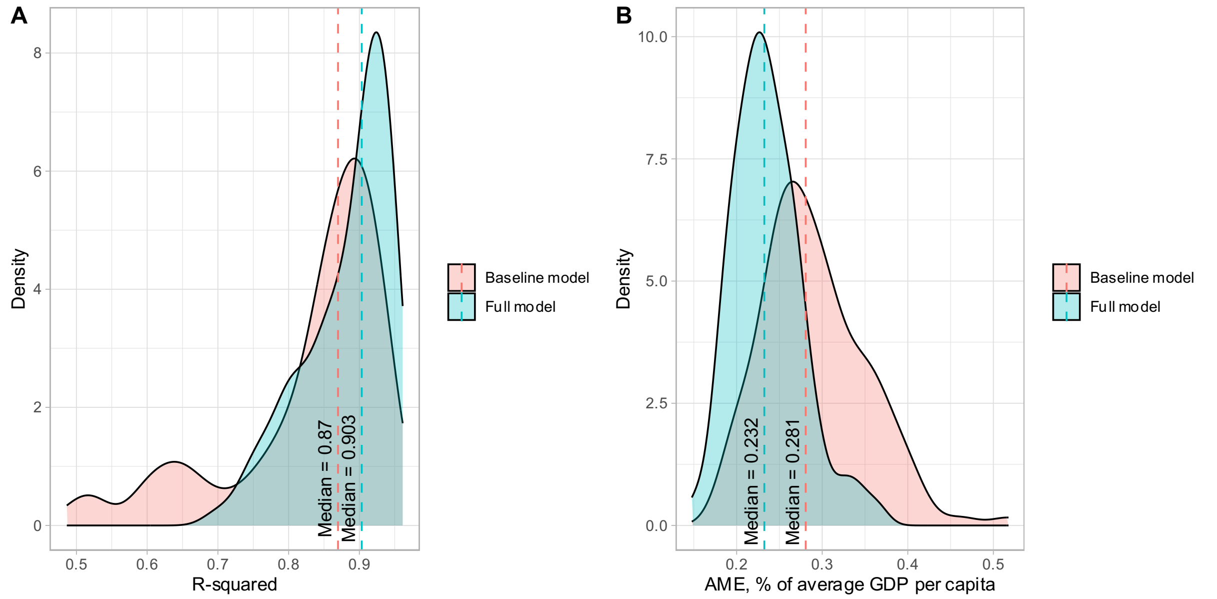

Figure S30. Model performance when not using dummies for supranational regions.

5.5.8. Predicting growth rates

An alternative to predicting GDP per capita levels is predicting GDP growth rates. We follow the same model setup to do so, but do not find positive results. Model performance metrics are significantly lower than when predicting GDP per capita levels, and there is no significant difference between the baseline model and the full model (see Figure S31).

We believe this is the case for two reasons. First, it is significantly harder to predict growth rates instead of levels. Second, we have a significantly lower amount of labeled training data. Specifically, we only have 455 true observations when predicting growth rates, while we can train our model on more than 1,300 observations when predicting levels.

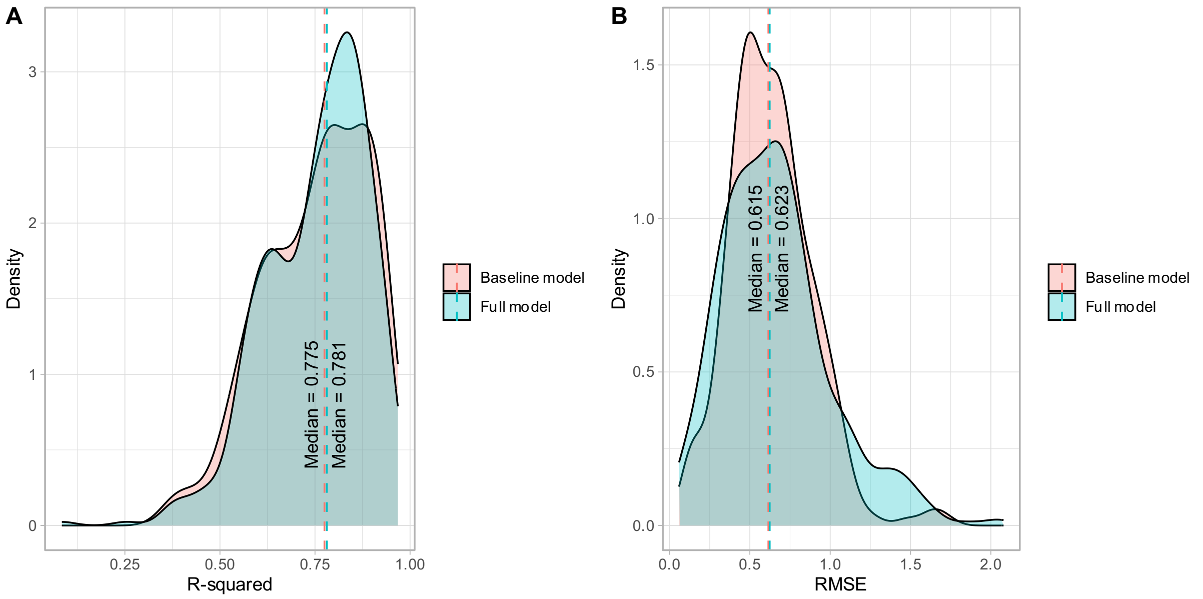

Figure S31. Model performance when predicting growth.

6. References

1. J. Bolt, J. L. van Zanden, Maddison style estimates of the evolution of the world economy. A new 2020 update. *Maddison-Proj. Work. Pap.* **WP-15** (2020).
2. J. Bolt, J. L. van Zanden, The Maddison Project: collaborative research on historical national accounts: The Maddison Project. *Econ. Hist. Rev.* **67**, 627–651 (2014).
3. F. Geary, T. Stark, Regional GDP in the UK, 1861-1911: new estimates: Regional GDP. *Econ. Hist. Rev.* **68**, 123–144 (2015).
4. Office for National Statistics, Historical Regional GDP 1968 to 1970 and 1971 to 1996. Office for National Statistics. Deposited 2016.
5. K. Enflo, M. Henning, L. Schön, "Swedish Regional GDP 1855–2000: Estimations and General Trends in the Swedish Regional System" in *Research in Economic History*, (Emerald Group Publishing, 2014), pp. 47–89.
6. K. Enflo, A. Missiaia, Regional GDP estimates for Sweden, 1571–1850. *Hist. Methods J. Quant. Interdiscip. Hist.* **51**, 115–137 (2018).
7. N. Delefortrie, J. Morice, Les revenus départementaux en 1864 et en 1954. *Population* **15**, 721 (1960).
8. M. P. Squicciarini, N. Voigtländer, Human Capital and Industrialization: Evidence from the Age of Enlightenment *. *Q. J. Econ.* **130**, 1825–1883 (2015).
9. E. Felice, The roots of a dual equilibrium: GDP, productivity, and structural change in the Italian regions in the long run (1871–2011). *Eur. Rev. Econ. Hist.* (2018). https://doi.org/10.1093/ereh/hey018.
10. C. Alvarez-Nogal, L. P. De La Escosura, The decline of Spain (1500-1850): conjectural estimates. *Eur. Rev. Econ. Hist.* **11**, 319–366 (2007).
11. M. Badia-Miró, J. Guilera, P. Lains, Reconstruction of the Regional GDP of Portugal, 1890 - 1980. *UB Econ. - Work. Pap.* **12/280** (2012).
12. E. Buyst, Reversal of Fortune in a Small, Open Economy: Regional GDP in Belgium, 1896-2000. *SSRN Electron. J.* (2009). https://doi.org/10.2139/ssrn.1586762.
13. Eurostat, Gross domestic product (GDP) at current market prices by NUTS 2 regions. Eurostat. Deposited 2023.
14. Office for National Statistics, Regional gross domestic product: all ITL regions. Office for National Statistics. Deposited 2022.
15. Bureau of Economic Analysis, Gross Domestic Product by Metropolitan Area. Bureau of Economic Analysis. Deposited 2018.
16. Statistics Canada, Metropolitan gross domestic product. Statistics Canada. Deposited 2014.
17. State Statistics Services Ukraine, Валовий регіональний продукт. Deposited 2013.
18. Belstat, Gross regional product at current prices. Deposited 2023.
19. Rosstat, Gross Regional Product at current basic prices per capita (1998-2019). Deposited 2020.
20. J. L. van Zanden, B. van Leeuwen, Persistent but not consistent: The growth of national income in Holland 1347–1807. *Explor. Econ. Hist.* **49**, 119–130 (2012).
21. S. N. Broadberry, B. M. S. Campbell, A. Klein, M. Overton, B. van Leeuwen, *British economic growth, 1270-1870* (Cambridge University Press, 2015).
22. N. Palma, J. Reis, From Convergence to Divergence: Portuguese Economic Growth, 1527–1850. *J. Econ. Hist.* **79**, 477–506 (2019).
23. P. Malanima, The long decline of a leading economy: GDP in central and northern Italy, 1300-1913. *Eur. Rev. Econ. Hist.* **15**, 169–219 (2011).
24. U. Pfister, Economic Growth in Germany, 1500–1850. *J. Econ. Hist.* **82**, 1071–1107 (2022).
25. M. Malinowski, J. L. van Zanden, Income and its distribution in preindustrial Poland.

Cliometrica **11**, 375–404 (2017).
26. C. Álvarez-Nogal, L. P. De La Escosura, The rise and fall of Spain (1270-1850). *Econ. Hist. Rev.* **66**, 1–37 (2013).
27. O. Krantz, Swedish GDP 1300-1560 : A Tentative Estimate. *Lund Pap. Econ. Hist. Gen. Issues* **152** (2017).
28. L. Ridolfi, Six Centuries of Real Wages in France from Louis IX to Napoleon III: 1250–1860. *J. Econ. Hist.* **79**, 589–627 (2019).
29. L. Schön, O. Krantz, New Swedish Historical National Accounts since the 16th Century in Constant and Current Prices. *Lund Pap. Econ. Hist. Gen. Issues* **140** (2015).
30. M. Laouenan, *et al.*, A cross-verified database of notable people, 3500BC-2018AD. *Sci. Data* **9**, 290 (2022).
31. A. Z. Yu, S. Ronen, K. Hu, T. Lu, C. A. Hidalgo, Pantheon 1.0, a manually verified dataset of globally famous biographies. *Sci. Data* **3**, 150075 (2016).
32. P. Bairoch, J. Batou, P. Chèvre, *La population des villes européennes de 800 à 1850* (Librairie Droz, 1988).
33. E. Buringh, The Population of European Cities from 700 to 2000: Social and Economic History. *Res. Data J. Humanit. Soc. Sci.* **6**, 1–18 (2021).
34. M. Schich, *et al.*, A network framework of cultural history. *Science* **345**, 558–562 (2014).
35. M. Serafinelli, G. Tabellini, Creativity over time and space: A historical analysis of European cities. *J. Econ. Growth* **27**, 1–43 (2022).
36. P. Koch, V. Stojkoski, C. A. Hidalgo, The Role of Immigrants, Emigrants, and Locals in the Historical Formation of European Knowledge Agglomerations. *Reg. Stud.* (2023). https://doi.org/10.1080/00343404.2023.2275571.
37. C. A. Hidalgo, Economic complexity theory and applications. *Nat. Rev. Phys.* **3**, 92–113 (2021).
38. D. Acemoglu, S. Johnson, J. Robinson, The Rise of Europe: Atlantic Trade, Institutional Change, and Economic Growth. *Am. Econ. Rev.* **95**, 546–579 (2005).
39. D. Acemoglu, D. Cantoni, S. Johnson, J. A. Robinson, The Consequences of Radical Reform: The French Revolution. *Am. Econ. Rev.* **101**, 3286–3307 (2011).
